# Self-assembled membrane composed of amyloid-like proteins for efficient size-selective molecular separation and dialysis

**DOI:** 10.1038/s41467-018-07888-2

**Published:** 2018-12-21

**Authors:** Facui Yang, Fei Tao, Chen Li, Lingxiang Gao, Peng Yang

**Affiliations:** Key Laboratory of Applied Surface and Colloid Chemistry, Ministry of Education, School of Chemistry and Chemical Engineering, Shaanxi Normal University, Xi’an, 710119 China

## Abstract

The design and scalable construction of robust ultrathin protein membranes with tunable separation properties remain a key challenge in chemistry and materials science. Here, we report a macroscopic ultrathin protein membrane with the potential for scaled-up fabrication and excellent separation efficiency. This membrane, which is formed by fast amyloid-like lysozyme aggregation at air/water interface, has a controllable thickness that can be tuned to 30–250 nm and pores with a mean size that can be tailored from 1.8 to 3.2 nm by the protein concentration. This membrane can retain > 3 nm molecules and particles while permitting the transport of small molecules at a rate that is 1~4 orders of magnitude faster than the rate of existing materials. This membrane further exhibits excellent hemodialysis performance, especially for the removal of middle-molecular-weight uremic toxins, which is 5~6 times higher in the clearance per unit area than the typical literature values reported to date.

## Abstract (web summary)

Membrane separation is important for a range of industrial and medical applications. Here, the authors report on the formation of self-assembled protein membranes for size selective separation and demonstrate application in the separation of dyes, nanoparticles and in hemodialysis.

## Introduction

**Fig. 1:**
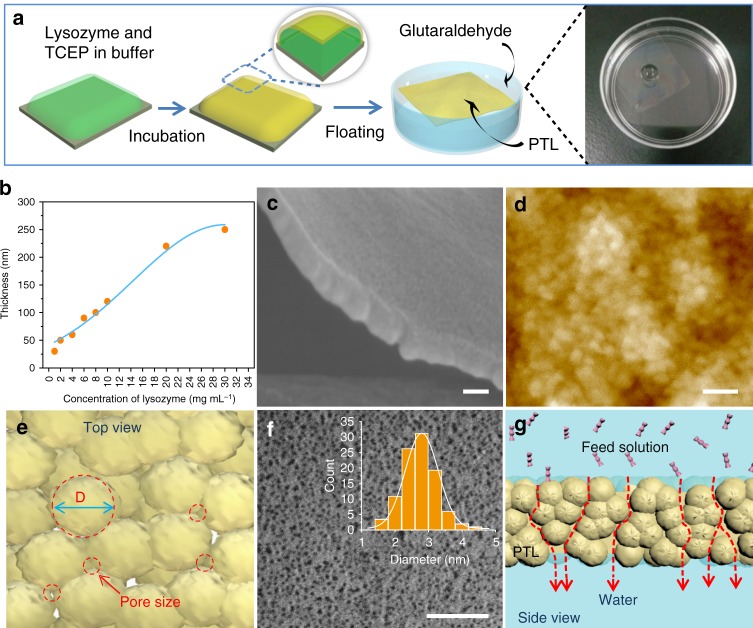
The cross-linked PTL membrane and its pore structures. **a** Schematic of the preparation steps of the cross-linked PTL membranes. **b** The relationship between the membrane thickness and concentration of lysozyme. **c** SEM image of the cross-section of the membrane. **d** AFM image of the membrane. **e** Top view of the membrane showing the pores resulting from the packing of oligomer particles. The pore diameter (*d*) and particle size (*D*) have a relationship of *d* = *D*(2−√3)/√3 = 0.1547D. **f** Freeze-fracture TEM image of the membrane and its pore size distribution. **g** Side view of the membrane showing oligomer-surrounded tortuous channels for molecular transport. Scale bars in **c**, **d**, **f** are 50 nm

Precise membrane separation is a ubiquitous and important technique^1–4^ that is widely utilized across the entire spectrum of chemical processes such as desalination^5,6^, protein separation and purification^7,8^, and hemodialysis^9–12^. In nature, biological membranes enable living systems to function without rapidly descending into a state of entropically driven chaos. Biological membrane separation is closely related to some functional proteins, typically ranging from passive water channels provided by aquaporin in the kidneys^13^ to sophisticated ion pump/channel proteins in heart tissue. In another simple yet elegant example, the 2–3 nm pores formed by adjacent glycoproteins in S-layers provide cells with an ideal physical barrier, which allows nutrients and metabolites to pass back and forth and protects the bacterium from threatening enzymes and phages outside the cell^14^. Unfortunately, the scaled-up production of biologically derived membranes towards the separation of a wide spectrum of molecules and particles has proved to be a formidable challenge^15–23^, which has led researchers and the membrane industry to focus on synthetic alternatives. Significantly, a range of fabrication procedures have been employed to synthesize protein-based membranes^19–22^. For example, free-standing ferritin membranes were prepared by guiding the fabrication with cadmium hydroxide nanostrands and subsequently removing these carcinogenic templates^22^. Robust membranes were also formed by hybridizing micro-/nanofibrils from silk fibroin or amyloidogenic β-lactoglobulin with inorganic fillers^20,21^. Given these recent advances in controlling the structure and morphology of protein-based membranes, the obstacles in this field are also obvious and typically involve the small membrane area restricted by the commonly used vacuum filtration-based fabrication^19–22^, the time- and cost-intensive pretreatments under harsh conditions required to obtain silk fibroin^19,20^ and amyloid fibrils^21^ (e.g., a reaction time of tens of hours with organic/inorganic additives, high temperature and strong acid), the use of toxic templates (e.g., cadmium hydroxide nanostrands to guide the assembly of ferritin)^22^ and polar solvents (e.g., hexafluoro-2-propanol to dissolve silk fibroin)^19,20^. In this regard, the facile and biocompatible construction of stable, ultrathin, pure protein-based membranes with selective gating for molecules and colloids is highly desirable and still remains as a great challenge. We previously found a reaction pathway for superfast amyloid-like protein assembly through the rapid reduction of the intramolecular disulfide bonds of lysozyme by tris(2-carboxyethyl)phosphine (TCEP)^24^. During this process, the α-helix structure of native lysozyme was rapidly unfolded and aggregated into β-sheet stacking-based oligomers. A free-standing protein nanofilm was then formed in ~2 h through the agglomeration of the oligomers at the air/water interface (Fig. 1a)^25^. Such a biomaterial, also called phase-transitioned lysozyme (PTL), is stable in various organic solvents (such as ethanol and hexane) and extreme pH conditions (pH 1-12) and exhibits good hemo-/cytocompatibility^26–28^.

In the present study, we report an economic, scalable and nontoxic approach to the fabrication of free-standing protein membrane, which is based on amyloid-like lysozyme oligomer aggregates; this approach does not require any doping additives and can be carried out under mild conditions (room-temperature and neutral aqueous solution). We reveal that pores with a mean size that can be tailored from 2 to 3 nm are spontaneously formed by adjacent amyloid-like lysozyme oligomers assembled as a nanomembrane at the air/water interface. This membrane with a controllable nanoscale thickness and large area (e.g., diagonal of 20 in.) readily supports fast dialysis at a transport rate that is 1–4 orders of magnitude faster than the rate of existing materials, allowing the universal and rapid separation of small molecules (e.g., dyes), middle molecules (e.g., toxins) and macromolecules (e.g., proteins) as well as nanoparticles. Remarkably, this membrane also exhibits excellent hemodialysis performance, especially for the removal of middle-molecular-weight uremic toxins, while maintaining a nearly 100% retention of serum proteins; this performance is 5–6 times higher in the clearance per unit area than the typical literature values.

## Results

### Pore size analysis of the free-standing PTL membrane

The mechanism for the formation of a PTL membrane was proposed previously: the superfast aggregation of lysozyme oligomers driven by β-sheet stacking results in the formation of a membrane at the air/water interface^24^. For membrane separation applications, the as-synthesized PTL nanofilm was further cross-linked with glutaraldehyde (e.g., 1 h at 37 °C) to enhance the mechanical robustness (Fig. 1a), and Fourier transform infrared (FTIR) analysis confirmed the cross-linking structure in the resultant PTL membrane (Supplementary Fig. 1). The functional groups on the membrane surface were characterized by X-ray Photoelectron Spectroscopy (XPS), which presented the structures on the membrane surface mainly including alphatic carbon (C-H/C-C), amines (C-N), hydroxyls (C-O), thiols (C-S), amides (O=C-N) and carboxyl groups (O=C-O) (Supplementary Fig. 2). These multiple functional groups were from hydrophilic and hydrophobic amino acid residues^27^, which provided unique and important uses^22,24–26^. These functional groups supported the co-contribution from ligand bond, electrostatic interaction, hydrogen bond, and hydrophobic interactions with metal, organic and inorganic material surfaces, and such a multiplex interfacial interaction model can provide the membrane with a universal interfacial bonding with virtually arbitrary material surface, regardless of chemical composition on material surfaces^27,28^. This feature is thus very useful to adhere the PTL membrane on virtually arbitrary membrane support to form a robust functional separation skin layer. These functional groups could also support sequential surface chemical derivatization and corresponding functionalization^25–28^. Unlike other preparation methods, which are restricted by time-consuming procedures and a small membrane area (e.g., vacuum filtration-based fabrication)^19–22^, in the fabrication method of PTL, the size of the PTL membrane could be simply controlled by adjusting the air/water interface area due to its interfacial formation feature^25^. At the laboratory scale, a film with a diagonal as large as 20 in. (0.14 m^2^) could be easily acquired in 2–4 h (Supplementary Fig. 3a). In general, the thinner the membrane is, the faster the diffusion rate through the membrane. In the present work, the membrane thickness could be tuned from 30 to 250 nm by simply varying the concentration of lysozyme (Fig. 1b and Supplementary Fig. 3b-i), which showed great potential for fast membrane separation. A PTL membrane with a thickness of 50 nm was typically selected for the following tests (Fig. 1c). By vertically supporting a static ethanol column, a test was carried out that revealed that a thicker membrane could withstand higher pressures. A pressure of 0.3–1.2 kPa was supported by a free-standing membrane with a thickness of 50–250 nm (Supplementary Fig. 4), which was comparable to the robustness of ferritin-based membranes (1.6 kPa)^22^. Such mechanical robustness ensured the membrane stability in the subsequent filtration process.

The oligomers assembled in the membrane had a diameter mainly centered at about 17–20 nm, as reflected by transmission electron microscopy (TEM) images (Supplementary Fig. 5a-f) and atomic force microscopy (AFM) (Fig. 1d). The hydrophobic interactions between protein molecules provided a driving force for their self-assembly into oligomer aggregates, and the water molecules from the surrounding hydrophobic side chains of protein molecules could then form the channels in the voids of the oligomer aggregates^22,29^. Actually, the weight percentage of internal water contained in the open volume of the cross-linked PTL membrane was measured as 10~11 wt% by thermogravimetric (TG) and differential scanning calorimetry (DSC) analysis (Supplementary Fig. 6). In the TG measurement (Supplementary Fig. 6a), the dried PTL membrane was utilized to evaluate the content of the internal water contained in the membrane. A mass loss (total 10 wt%) due to moisture evaporation from internal adsorbed water was observed from room temperature until 100 °C (boiling point of water at normal pressure). The membrane was then saturated with water and analyzed by DSC (Supplementary Fig. 6b), the small but sharp endothermic peak found at −0.7 °C in the DSC curve, which was assigned to the melting of freezable surface-bound water in the pores of the membrane. Such melting temperature (−0.7 °C) was lower than the normal fusion temperature (0 °C) of ice, and this phenomenon was because that the freezing process of the surface-bound water competed with the water-material surface interaction^30^. From the enthalpy change calculated by the area under the DSC peak at −0.7 °C^22^, the content of surface-bound water (filled water in the pores of the membrane) was thus estimated to be 11 wt%, which was close to the internal water content (10 wt%) estimated by the TG curve. Based on the internal water content in the membrane, the porosity of the membrane was thus evaluated within 10–11% with the supposition that the density of proteinaceous material was close to the water density. For 50-nm-thick nanomembrane, the surface porosity is also useful that was further estimated as 3.2% by using the Hagen-Poiseuille equation (Equation 4 in Methods), since this equation has been widely applied to the filtration membranes with flow channels larger than 2 nm in diameter^22^. An interfacial channel surrounded by three such oligomers with a diameter ~20 nm could be geometrically calculated to have a size of 3.1 nm^31^ (Fig. 1e). This value was nearly consistent with the measured pore size of ~2.9 nm in diameter, as revealed by the freeze-fracture TEM bright-field image of the membrane (Fig. 1f). Compared with the theoretical value, the measured pore diameter was slightly decreased, which may be attributed to a denser packing structure after crosslinking with glutaraldehyde. As a large number of such voids existed among the oligomers, the molecules or ions could then be transported along the interconnected tortuous channels, which thus constructed the foundation for membrane separation (Fig. 1g).

### Pore size measurements based on the rejection of linear polymers

**Fig. 2:**
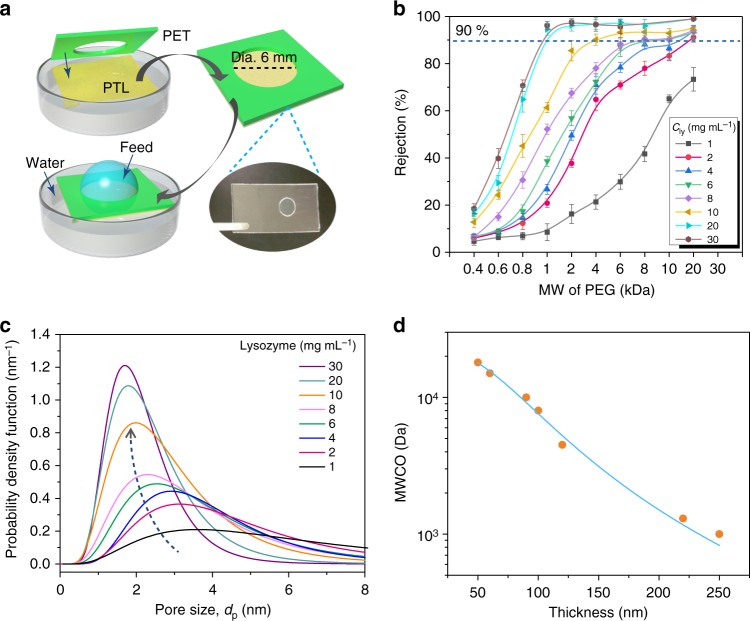
The pore size of the cross-linked PTL membranes and rejection of neutral linear PEG. **a** Schematic of the permeation of PEG dissolved in water. The membrane was transferred to a PET substrate with a 6 mm dia. pore size for the permeation tests. **b** Rejection of PEGs of different molecular weight by membranes of different thickness (as controlled by the lysozyme concentration during the membrane preparation). Three repeated tests were performed for each set of measurements and error bars represent standard deviation. **c** Probability density function curve showing the pore size distribution of the cross-linked PTL membrane. The lines represent the fitting to the solutions of the simultaneous differential equations in Equation (2). **d** The molecular weight cut-off (MWCO) of the membranes with different thicknesses

The pore size of the membrane was further evaluated by a permeation assay with neutral polyethylene glycol (PEG) molecules of different molecular weight. For this purpose, the membrane formed at the air/water interface was transferred to a polyethylene terephthalate (PET) support with a hole (dia. 6 mm) to control the effective permeation area (Fig. 2a). The membrane could be stably adhered onto the PET substrate due to the multiplex interfacial bonding of the membrane with a material^28^. The PET-supported membrane was floated on the pure water surface, and then 50 μL of feed PEG solution was dropped onto the membrane surface to probe the PEG permeation into the water within 12 h. The Einstein-Stokes radius (ESR) for PEG is closely correlated with its molecular weight^32^, and the higher the molecular weight is, the larger the molecular ESR (Supplementary Fig. 7). For a membrane with a given thickness, the rejection ratio increased with an increasing molecular weight of PEG due to stronger channel tortuosity on larger polymer chains (Fig. 2b, Equation 1). The pore size of the membrane was then defined as the ESR of PEG when its rejection ratio through the membrane reached 90%, and the corresponding molecular weight was denoted as the molecular weight cut-off (MWCO) of the membrane^32^. As the membrane thickness increased from ~30 to ~250 nm controlled by the lysozyme concentration (from 1 to 30 mg mL^−1^) (Fig. 1b), the pore size distribution of the membrane gradually narrowed, with the centered diameter shifting from 3.6 to 1.8 nm (Fig. 2c, Equation 2). The corresponding MWCO for the polymer transport through the membrane was reasonably estimated at 18–1 kDa (50–250 nm in thickness), which was tailored effectively by the membrane thickness (i.e., the pore size) (Fig. 2d). Compared with frequently used polymer membranes typically generated by electrospinning^33,34^, the PTL membrane displayed good uniformity with easily tunable distributions of the pore size and MWCO. The diffusion rate analysis further reflected that the transport of PEG through the membrane was obviously controlled by the molecular weight (size) and diffusion time. With the increase of molecular weight of PEG, the penetration rate was decreased, and the penetration of PEG with different molecular weight through the membrane required no more than 12 h to reach an equilibrium concentration in the permeated solution (Supplementary Fig. 8a, b). It was further found that the non-specific adsorption of PEG on the membrane was low (Supplementary Fig. 8c), and the subsequent continuous penetration of PEG with different molecular weight using these membranes showed a stable rejection performance in repetitive diffusion tests for at least 20 cycles (Supplementary Fig. 8d). The PEG solutions used in the present work had the highest concentration around 0.05% (w/v), which was well below the overlap concentration (30%) for PEG in a semidilute regime^35–37^. For the present system, on the one hand, PEG chains with a characteristic size being much smaller than the pore diameter, would easily enter into the pores and pass through the membrane; on the other hand, large PEG chains are excluded from the pores because of entropic or steric effects.

The ESR of PEG-based pore size distribution of the membrane was further verified by the test of nitrogen adsorption/desorption isotherms (Supplementary Fig. 9). From this test for the PTL membranes with 50, 100, and 250 nm in thickness, it could be seen that the pore diameter was decreased from 2.8 to 1.6 nm with the increase of the membrane thickness from 50 to 250 nm. The total Brunauer–Emmet–Teller (BET) surface area was decreased from 14.2 to 4.7 m² g^−1^, together with steady contraction of the total Barrett–Joyner–Halenda (BJH) pore volume from 0.05 to 0.02 cm³ g^−1^, as the membrane thickness increased from 50 to 250 nm. For all of 50-, 100-, and 250-nm-thick membranes, the pore size distribution from the test of nitrogen adsorption/desorption isotherms showed the same overall trend as that from the ESR of PEG-based experiments.

### Size-selective molecular diffusion by the PTL membrane

**Fig. 3:**
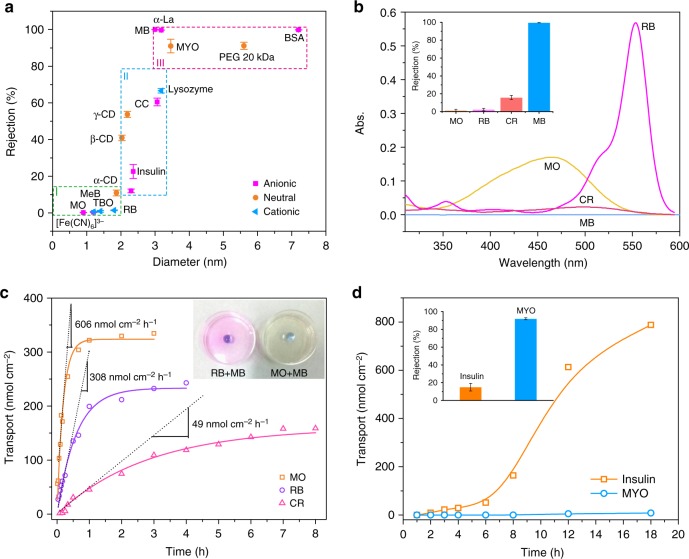
The separation performance of the cross-linked PTL membranes. **a** Separation performance of the 50-nm-thick PTL membranes for neutral and ionic compounds including region I, <10% rejection of molecules with diameters <2.0 nm; region II, 20–80% rejection of molecules with diameters of 2–3 nm; and region III, >90% rejection of molecules larger than 3 nm in diameter. **b** UV-vis spectra of the water side below the membrane after 12 h of permeation by using different dyes as the feed solution and the corresponding rejection ratios (inset). **c** Time-dependent transport of dyes through the PTL membrane and the transport rate calculated as the initial slope of the transport curve^7,38^. Optical photographs to show how the membrane rejects large molecules (methyl blue, MB) while allowing small molecules (methyl orange, MO and rhodamine B, RB) to pass through. **d** Time-dependent transport of proteins through the PTL membrane and the rejection ratios of MYO and insulin from the mixture (inset). The initial concentration of the protein solution is 10 mg mL^−1^. Data shown are the average of triplicates with standard deviation

By using a series of molecules ranging from 1.2 to 7.2 nm in size (0.1–100 kDa in molecular weight), we systematically probed the effect of molecular size/shape on the permeability through the 50 nm-thick membrane with a critical pore size of 3 nm (all tested chemicals are listed in Supplementary Table 1). By dropping the feed solution on the membrane floating on the pure water surface (Fig. 2a), a diffusion gradient of molecular transport from the feed solution (high concentration) to the pure water side was created^38^ to control the molecular permeation through the membrane. The entire separation diagram could be divided into three regions (Fig. 3a). In region I, where the target molecules for probing were smaller than 2 nm, the molecules could easily diffuse through the membrane with a rejection ratio lower than 10%. In region II, where the molecular size was ~2–3 nm, which is close to the critical pore size of the membrane, the molecules could achieve a moderate rejection ratio, e.g., from 20 to 80%, through the membrane. In region III, where the molecular size was higher than 3 nm, the molecules were largely blocked by the membrane, with a typical rejection ratio higher than 90%^7,39,40^.

The ability of the membrane to separate small molecular dyes dissolved in water was explored first^41^. While methyl orange (MO, 1.2 nm), methylene blue (MeB, 1.2 nm), toluidine blue O (TBO, 1.4 nm) and rhodamine B (RB, 1.8 nm) passed through the membrane with a nearly zero rejection ratio, congo red (CR, 2.3 nm) was transported across the membrane with a rejection ratio of approximately 12 ± 1% (mean ± standard deviation) due to its relatively larger size than MO and RB (Fig. 3a). In contrast, the membrane blocked methyl blue (MB, 3 nm) with a 99.6% rejection ratio either from a pure solution of MB (Fig. 3a) or mixed solutions of MB and MO or MB and RB (Fig. 3b). The PTL membranes are expected to be highly efficient in molecular separation because they are thin and have a minimal filter surface area^7^. Based on the sizes of the target solutes, from 1.2 (MO) to 2.3 nm (CR), the membrane exhibited an initial transport rate of 606 nmol cm^−2^ h^−1^ for MO, 308 nmol cm^−2^ h^−1^ for RB and 49 nmol cm^−2^ h^−1^ for CR (Fig. 3c, Equation 3). These rates were more than 1-3 order of magnitude faster than those of thick nanofabricated membranes^42^ (e.g., alumina membranes, Yobios dialysis membrane with 1 kDa MWCO) and >4 times faster than those of ultrathin porous nanocrystalline silicon membranes^7^. The 100% rejection ratio of MB was much higher than that of other molecules with a similar size to MB (e.g., cytochrome C and lysozyme) (region II of Fig. 3a), which reflected a shape-directed effect on the molecular separation^22^, especially when the target molecule had a molecular size close to the critical pore size of the membrane. In contrast to the nearly linear MO, RB, MeB, TBO and CR, MB is a branched aromatic heterocyclic compound (Supplementary Fig. 10), and its structure would not allow enough flexibility for MB to adaptively pass through the membrane. Such separation performance was not deteriorated by using the membrane treated with acidic (pH 1) or alkaline (pH 12) aqueous solution (Supplementary Fig. 11a-c), which indicated a good stability of the membrane towards an extreme pH range (typically, 1–12). Such separation performance was also not the result of simple non-specific adsorption of dyes on the membrane surface, because it was found that MB, MeB, MO, and TBO were only adsorbed on the membranes with a low extent (Supplementary Fig. 12a–d). The low adsorption of dyes on the membrane reflected that the hydrophobic and H-bonding interaction between the dyes and native lysozyme^43^ may not be predominantly existed between the dyes and the PTL membrane containing amyloid-like lysozyme aggregates from partially unfolded proteins. The low adsorption of dyes on the membrane further guaranteed the repetitive separation of MB from a dye mixture through the same membrane for at least 10 times cycles (Supplementary Fig. 12e, f). In contrast to the shape-directed effect on the molecular rejection for small molecules (e.g., MB), a conformational change might be possible for flexible biopolymers (e.g., cytochrome C and lysozyme) to result in an enhanced permeation ratio (region II of Fig. 3a). Such steric effect was also reflected by the separation difference between linear PEG and large-ring cyclodextrin (CD) (Supplementary Fig. 13a–d)^22^. As the structure changed from α- to β- to γ-CD, the rejection ratio through the 50-nm-thick membrane increased, and the value for the largest structure, γ-CD (2.2 nm), was 53.7 ± 1.6% (Supplementary Fig. 13e). In comparison, the rejection of PEG with a similar molecular size (2.3 nm) to γ-CD by the membrane only reached 27.7 ± 1.4%. This difference implied that during the transport of PEG through the membrane pores, the flexible PEG chain might be deformed in the pores, facilitating molecular permeation. In contrast, the rigid CD ring could not effectively undergo adaptive molecular deformation to fit into the pores, which thus led to a relatively high rejection ratio.

Based on model proteins including insulin (2.4 nm), cytochrome C (CC, 3.0 nm), lysozyme (3.1 nm), α-lactalbumin (α-La, 3.1 nm), myoglobin (MYO, 3.5 nm), and bovine serum albumin (BSA, 7.2 nm)^44^, the membrane further showed effective separation of proteins according to their molecular size, which may become the foundation for versatile biomedical applications such as hemodialysis treatment. In general, the rejection ratios for all of the tested proteins were elevated by increasing the membrane thickness (e.g., from 30 to 90 nm), which was controlled by the lysozyme concentration (e.g., from 1 to 4 mg mL^−1^) used in the membrane preparation (Supplementary Fig. 14a). For a typical 50-nm-thick membrane, high rejection of proteins larger than the critical pore size (3 nm) was obtained, including 90.6 ± 3.1% for MYO (3.5 nm) and 98.4 ± 1.4% for BSA (7.2 nm). Such separation performance was also not the result of simple non-specific adsorption of proteins on the membrane surface, since it was found that BSA (as a model protein) with fluorescein isothiocyanate conjugated (BSA-FITC) was only adsorbed on the membranes with a low extent (Supplementary Fig. 12a-d). The size selectivity was further demonstrated by separating MYO effectively from a mixture of insulin and MYO, which differ in size by approximately 1 nm (Fig. 3d and Supplementary Fig. 14b). The time lag for the breakthrough of insulin was approximately 4 h, while the transport for MYO was in a nearly complete block. The electrostatic attraction between the target solute and the PTL membrane may also play a role in the separation, especially when the target molecule has a molecular size that is close to the critical pore size of the membrane. For example, the PTL membrane had a positive zeta potential under neutral or acidic pH conditions^25^, which preferentially captured negatively charged α-La (pI 4.7) with a rejection ratio of 97 ± 0.6%, while the rejection ratio for positively charged lysozyme (pI 11.0) with a similar size to α-La was 65 ± 1.3%.

### Hemodialysis performance of the PTL membrane

The existence of water channels in the PTL membrane was then utilized to develop a forward osmosis (FO) process^45^. During the molecular separation (e.g., PEG) through the membrane, it was observed that water was also transported from the pure water side (low osmotic pressure) to the feed solution side (high osmotic pressure). Noticeable water transport from the pure water side to the salt solution side (1 M MgCl_2_) was further observed in 1 h (Supplementary Fig. 15a). The corresponding water flux was enhanced by decreasing the membrane thickness, and for a typical 50-nm-thick membrane, the water flux was 14.2 ± 1.6 L m^−2^ h^−1^ without applied pressure (Supplementary Fig. 15b), which is comparable to the existing commercial cellulose triacetate (CTA) FO membrane (9 L m^−2^ h^−1^)^46^. The FO process was then systematically studied by using a series of salts as the draw solution. For this aim, a diffusion setup to assess the water transport across the membrane was designed, in which the PTL membrane supported by a PET nuclear pore membrane (PTL/PET) was mounted between two cells (Supplementary Fig. 16a-e). After 12 h, a difference in the liquid level between the two cells was observed due to water transport from the pure water side to the electrolyte solution side (Supplementary Fig. 16a). The water transport flux was largely affected by the type of electrolyte dissolved in the draw solution, showing a gradual increase following an order of KCl < NaCl < CaCl_2_ < CuCl_2_ < MgCl_2_ (Supplementary Fig. 16f). With the same anion in the draw solution (Cl^−^), this order was consistent with the increase in cationic radius, i.e., K^+^ (0.331 nm) < Na^+^ (0.358 nm) < Ca^2+^ (0.412 nm) < Cu^2+^ (0.419 nm) < Mg^2+^ (0.428 nm), and thus attributed to a slower transfer rate for larger cations across the membrane^47,48^. This osmotic pressure-driven permeation allowed the PTL membrane to be operated in an FO mode with low energy consumption and fouling propensity compared to reverse osmosis membrane technology^13,45^.

**Fig. 4:**
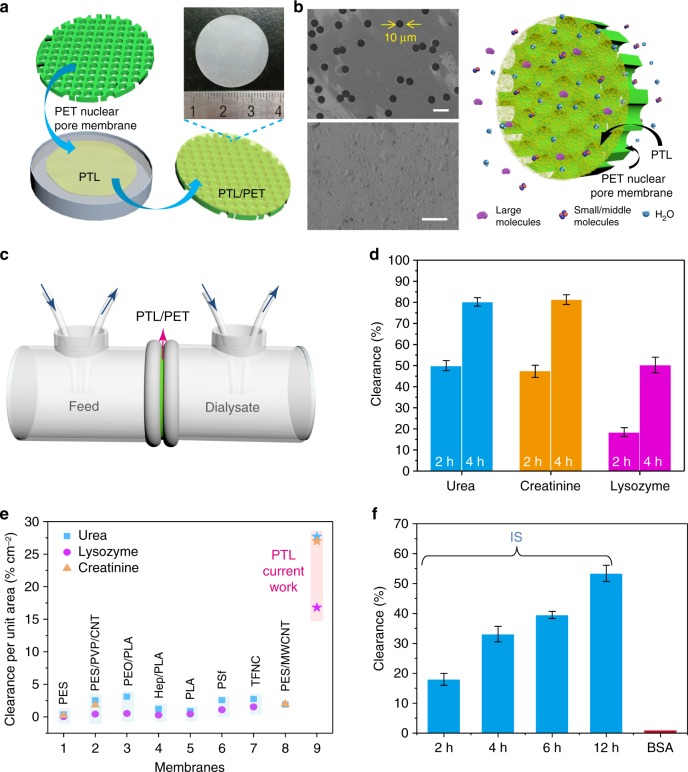
The dialysis performance of the PTL membrane. **a** Schematic of the preparation of the PTL/PET membrane and an optical photograph of the PTL/PET membrane. **b** SEM images of the PET nuclear pore support before (top) and after (bottom) it was covered by the PTL membrane, scale bars, 20 μm, as well as a schematic of an ideal hemodialysis membrane (removes excess water and toxic metabolites and prevents the loss of necessary proteins) with an active PTL separation layer and a scaffold-like PET support. **c** The dialysis setup including the simulated solution for dialysis on the feed side and pure water on the dialysate side. **d** Size-selective separation of uremic toxins by the PTL/PET membrane: urea, lysozyme and creatinine. **e** Comparison of the clearance ratio per unit membrane area of the PTL/PET membrane with typical literature values reported to date^49–53^. **f** The removal of indoxyl sulfate by the PTL/PET membrane at different time. Data shown are the average of triplicates with standard deviation

The above studies were further extended to an application for hemodialysis (HD) treatment (Fig. 4a–c). HD is the main means for treating patients with renal diseases. The key focus of HD is to remove small water-soluble uremic toxins (<500 Da) and middle-molecular-weight uremic toxins (500–20000 Da) while retaining albumin, which is large, in the blood to a maximum extent (Fig. 4b). Currently, the membranes for HD treatment are mainly based on the use of synthetic polymers^11,49^ and are limited in their ability to remove molecules of intermediate size (e.g., β_2_-microglobin, 11.8 kDa) from the blood. As a result, it is still a major obstacle to produce biologically derived membranes with good biocompatibility and removal efficiency for middle-molecular-weight toxins. Lysozyme (14 kDa), a frequently used model of intermediate-size molecules in HD tests^49^, was added into a simulated solution for HD treatment that contained urea, creatinine, lysozyme, and BSA in ultrapure water. After mounting the PTL/PET (area of 3 cm^2^) into the setup (Fig. 4b, c), the simulated mixed solution was circulated at a flow rate of 10 mL min^−1^ on the feed side at ambient temperature. A total of 82.2 ± 2% of the urea was cleaned out of the solution after 4 h with a dialysate flow rate of 10 mL min^−1^ (Fig. 4d and Supplementary Fig. 17a). Simultaneously, the membrane exhibited nearly 99.7% BSA retention, 50.3 ± 3.7% lysozyme clearance, and 81.3 ± 2.3% creatinine clearance (Fig. 4d and Supplementary Fig. S17b-d). As shown in Fig. 4e, the clearance ratio per unit membrane area (% cm^−2^) of the PTL membrane was 5-6 times higher than the typical literature values reported to date (Fig. 4e)^49–53^, indicating good efficiency of the PTL membrane in the removal of urea, lysozyme and creatinine. Conventional membranes for HD treatment typically comprise a relatively thick polymer layer with tortuous pores, which might not facilitate the easy passage of middle-molecular-weight uremic toxins through the membrane. In contrast, the above results demonstrated that lysozyme could pass easily through the ultrathin PTL membrane, resulting in high clearance efficiency, while large molecules such as albumin were completely blocked.

Beside middle molecules, protein-bound uremic toxins (PBUTs), particularly indoxyl sulfate (IS)^54^, are strongly bound to proteins and difficult to remove with the conventional HD treatment. An aqueous mixture of IS (25 mg L^−1^) and BSA-FITC (1.0 mg mL^−1^) with concentrations similar to those in actual samples was chosen in a dialysis simulation experiment^55^. The results showed that 33.1% of the IS was cleaned out of the solution after 4 h of circulation, which was comparable to the typical literature results for the standard HD treatment (31.8%) at the same dialysis duration^56^. For the PTL membrane, this value could be further improved by a prolonged dialysis time. For instance, the rejection ratio for IS increased to 53.4% by prolonging the dialysis time to 12 h, while BSA-FITC was hardly removed from the feed solution during the entire dialysis process (Fig. 4f and Supplementary Fig. 18a, b).

### The compatibility with pressure-driven filtration

As a newly discovered proteinaceous membrane, the adaptability of the PTL membrane to common pressure-driven filtration was further evaluated. Pressure-driven filtration can be scaled up easily, allowing fast separation and purification of various molecules/nanoparticles at an industrial scale^57^. Similar to the above dialysis setup, the PTL membrane, as an active skin layer, was adhered onto a PET nuclear pore membrane for pressure-driven filtration. In such a process, PET nuclear pore membrane (12 μm in thickness with a pore diameter of 10 μm) as a supporting frame possessed large cylindrical pore structure, which thus provided direct paths for the water and molecules after they penetrated through the ultrathin PTL membrane. The resultant water flux was inversely correlated to the thickness of the PTL membrane, and a high water flux of 226 ± 17 L m^−2^ h^−1^ could be easily achieved by a typical 50-nm-thick PTL membrane at a low pressure difference of 100 kPa (Supplementary Fig. 19). This value was 4–20 times higher than the fluxes that can be withstood by several commercial filtration membranes with similar rejection properties^6^. In the SEM image of the PTL membrane after pressure-driven filtration, it was clearly visible that the membrane was not damaged (Supplementary Fig. 20a-d).

**Fig. 5:**
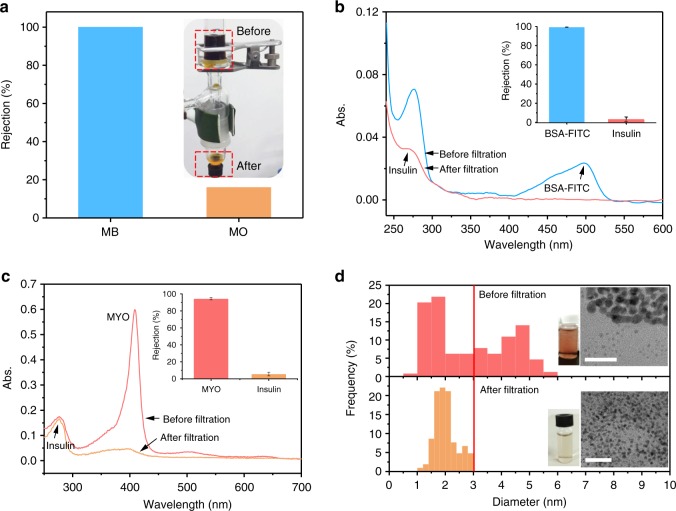
Pressure-driven filtration through the PTL membrane. **a** Rejection ratios of MB and MO from a dye mixture and the corresponding setup (inset). **b** UV/Vis spectra and rejection ratios (inset) of a protein mixture (BSA-FITC and insulin) before and after the filtration. **c** UV/Vis spectra and rejections ratios (inset) of a protein mixture (MYO and insulin) before and after the filtration. **d** TEM images and corresponding diameter distributions of gold nanoparticles in the feed solution before (1.0–6.0 nm) and after the filtration (1.0–3.1 nm). Scale bars are 20 nm. Data shown are the average of triplicates with standard deviation

Pressure-driven filtration through a typical 50-nm-thick PTL membrane was then utilized to separate mixed organic dyes in aqueous solution, showing ~100% rejection ratio of MB in a mixed solution of MB/MO dyes (Fig. 5a and Supplementary Fig. 21a). The separation stability of the membrane was further evaluated according to the deterioration extent of the dye separation performance after swelling the membrane in ethanol^58^. After 12 h of immersion in ethanol, the membrane was taken out and washed with DI water completely. The pressure-driven filtration of the mixed dyes through the ethanol-treated membrane was then measured, and the rejection ratios were similar to those for the membrane before immersion in ethanol^25^ (Supplementary Fig. 22).

Protein separation was further achieved by pressure-driven filtration. A rejection ratio of BSA of ~100% was achieved from an aqueous solution of BSA and insulin (Fig. 5b and Supplementary Fig. 21b), and a rejection of MYO of ~90% was obtained from an aqueous solution of MYO and insulin (Fig. 5c). For a mixture of BSA-FITC and lysozyme, the separation of BSA was maintained at approximately 100% with a moderate rejection ratio of lysozyme (40.6 ± 9.3%) (Supplementary Fig. 21c). Prompted by the above results, this membrane was further applied to remove bilirubin (2.9 nm in diameter) at a rejection ratio of ~50% from a solution containing BSA and bilirubin (Supplementary Fig. 21d). Bilirubin, one of the breakdown products of red blood cell degradation, is a typical toxin in the blood, and the effective clearance of bilirubin is important for the detoxification of blood^59^. Compared with existing bilirubin adsorbents, this biocompatible membrane is advantageous for bilirubin removal due to its short diffusion path, low pressure drop and simple scaled-up process.

The water contact angle of the membrane was recorded as 70° and the surface roughness parameter (root mean square, RMS) was 2.5 nm on the membrane surface, as characterized by AFM. The relative hydrophilicity and smooth surface were favorable to the resistance to fouling^25,60^. The fouling extent of the PTL membrane was further evaluated by static protein adsorption on the membrane. On the basis of the low adsorption of BSA (Supplementary Fig. 12a-d), the membrane was further challenged by exposing it in 5 mL solution of BSA-FITC with a concentration of 100 mg L^−1^ for 6 h at 27 °C. Since the membrane was positively charged^25^, the membrane may adsorb BSA-FITC via electrostatic interaction at a low extent, as shown by the fluorescent microscopy (Supplementary Fig. 23a, b). The adsorbed protein on the membrane could be then desorbed in 3 M guanidine hydrochloride aqueous solution for 6 h (Supplementary Fig. 23c), and the re-cleaned membrane could be re-used for the separation of BSA-FITC and insulin, which showed the effective separation without obvious performance deterioration for at least 10 cycles by the pressure-driven filtration (Supplementary Fig. 23d). The low fouling extent was also supported by the low dye adsorption on the membrane (Supplementary Fig. 12a–d). Among all the tested dyes, MO showed the highest adsorption ratio on the membrane (~0.5%), because MO as a negatively charged molecule may be adsorbed onto the positively charged membrane surface via electrostatic interaction. Similar to the regeneration of the BSA-adsorbed membrane, it was then easy to desorb the adsorbed MO by using protic acid (e.g., the immersion in 0.01 M HCl for 4 h) to regenerate the membrane (Supplementary Fig. 23e). The reusability of the membrane was then verified by the pressure-driven filtration of the mixed dye solution (250 mg L^−1^  MO and 250 mg L^−1^ MB) through the regenerated membrane for at least 10 cycles (Supplementary Fig. 23e). The above results thus reflected that although the PTL membrane may have a low but non-zero fouling extent, it could be re-used to overcome effectively such disadvantage by simple rinsing (regeneration).

In addition to molecules, the membrane was also able to selectively separate nanoparticles of different sizes by the pressure-driven filtration. A mixed solution of gold nanoparticles with sizes ranging from 1.0 to 6.0 nm was filtered through the typical 50-nm-thick membrane under pressure. Since the membrane was not a single layer but stacked by a multilayer of protein oligomers (Fig. 1g) and around 10% pores in the PTL membrane were in the size about 3.5 nm (Fig. 1f), so that the large Au nanoparticles with a diameter being a little higher than the pore size would be blocked by the tortuous channels among the oligomers. The size measurements of Au nanoparticles by both of TEM and dynamic light scattering (DLS) then demonstrated that the membrane blocked effectively the penetration of Au nanoparticles with a DLS size higher than 3.7 nm (Supplementary Fig. 24) or a TEM size higher than 3.1 nm (Fig. 5d) by the pressure-driven filtration.

## Discussion

We have proposed a facile and template-free strategy for the low-cost, environmentally friendly and rapid synthesis of a large-area (e.g., diagonal of 12 in.), free-standing protein membrane (e.g., formation in 2–4 h) that consists of a densely packed structure of amyloid-like lysozyme oligomers. In contrast to the known biologically derived membranes, pores with a mean size that can be tailored from 2 to 3 nm are spontaneously formed by adjacent amyloid-like lysozyme oligomers in the PTL membrane. The membrane is robust enough to sustain molecular size-directed diffusion and pressure-driven filtration, exhibiting a universal separation ability for small molecules (e.g., dyes), middle molecules (e.g., toxins), macromolecules (e.g., proteins) and a diffusion rate that is 1–4 orders of magnitude faster than the rate of existing materials. The PTL membrane exhibits excellent hemodialysis performance, especially for the removal of middle-molecular-weight uremic toxins, while maintaining a nearly 100% retention of serum proteins; this performance is 5–6 times higher in the clearance per unit area than the typical literature values reported to date. This work underlines the importance of amyloid-like protein oligomers in the scaled-up preparation of biomimetic membranes. The application of this robust, ultrathin protein-based membrane with efficient sieving of molecules and colloids to versatile separation devices at the macro- and microscale (e.g., size-directed forward osmosis, large-scale dialysis system and pressure-driven filtration) and other areas of science and technology is very promising.

## Methods

### Materials and chemicals

Lysozyme was purchased from Sigma-Aldrich. Tris (2-carboxyethyl) phosphine hydrochloride (TCEP) was purchased from TCI. Cyclodextrin (α-CD, β-CD, γ-CD), cytochrome C, bilirubin, methyl orange, congo red, rhodamine B, methyl blue, methylene blue, toluidine blue O, indoxyl sulfate potassium salt, creatinine, insulin and myoglobin were purchased from Aladdin. HEPES (4-(2-hydroxyethyl)-1-piperazineethanesulfonic acid) buffer (pH = 7.2–7.4), bovine serum albumin (BSA) and BSA with fluorescein isothiocyanate conjugated (BSA-FITC) were obtained from Solarbio. Hydrochloric acid (HCl), polyethylene glycol (PEG), urea, sodium hydroxide (NaOH), sodium chloride (NaCl), sulfuric acid (H_2_SO_4_), absolute alcohol and acetone were purchased from Sinopharm Chemical. Track-Etched polyethylene terephthalate (PET) nuclepore membranes were purchased from Wuwei Kejin Xinfa technology Co. Ltd. (Gansu, China). Ultrapure water was used in all experiments and was supplied by Milli-Q Advantage A10 (Millipore, USA).

### Preparation of the cross-linked PTL membrane

The two types of membranes used in this study were a free-standing cross-linked PTL membrane and a cross-linked PTL membrane supported by a PET nuclear pore membrane (PTL/PET). The phase transition buffer containing lysozyme was freshly prepared by mixing a stock buffer solution of lysozyme (2 mg mL^−1^ in 10 mM HEPES buffer at pH 7.2) with TCEP buffer (50 mM TCEP in 10 mM HEPES buffer at pH 7.0, pH adjusted by 5 M NaOH) at a volume ratio of 1:1. The protein phase transition buffer was dropped on a piece of glass (e.g., 18 × 18 mm). Then, the solution on the substrate was incubated in a humid environment (generally for 50~240 min) at room temperature. The phase transition of lysozyme was initiated spontaneously upon mixing, and the PTL product in the form of a nanomembrane was formed at the solution surface. Then, the PTL nanomembrane was cross-linked by submerging it in 1 wt% aqueous glutaraldehyde for 1~6 h and rinsing it in water. For the PTL/PET membranes, the prepared cross-linked PTL membranes were adhered directly to the nuclear pore membranes.

### Membrane characterization

All SEM observations of the membrane thickness were carried out using a field emission scanning electron microscope (SU8020, Hitachi) at an acceleration voltage of 1 kV, and all the samples were not coated with gold. AFM was used to assess the surface topography of the membrane on a Dimension Icon atomic force microscope (Bruker) in ScanAsyst mode and a CSPM 5500 instrument (MultiMode, NanoScope IV from Benyuan Inc., China) in tapping mode. The morphology and structure of the membranes were characterized by field emission TEM (Tecnai G2 F20) at 200 kV without staining, and freezing-etching TEM samples were prepared using a high-vacuum evaporator HUS-5GB (Hitachi, Japan). FTIR spectra (the transmission mode with the use of the KBr pellet) were obtained between 400 and 4000 cm^−1^ with a resolution of 1 cm^−1^ using an Alpha-T spectrometer (Bruker). Thermogravimetry analysis was conducted by a thermogravimetric analyzer (Q50 TGA, TA) at 10 °C min^−1^. XPS was obtained with AXIS ULTRA from Kratos Analytical Ltd., the binding energies were calibrated by setting C1s peak at 284.6 eV. Particle size distribution of gold nanoparticles was performed by DLS on a Malvern Zeta sizer Nano-ZS90. The UV-Vis spectra of the membrane were measured by using UV-3600 spectrophotometer (Shimadzu).

### Molecular diffusive separation through the cross-linked PTL membrane

In all molecular penetration experiments, the volume of solutions was maintained at 50 μL, and the concentration of each species was 0.5 mg mL^−1^. The cross-linked PTL membrane with an effective area of 0.28 cm^2^ was floating at the surface of 5 mL of water, and the permeation into the water was then characterized by ultraviolet-visible (UV-vis) spectroscopy (U-3900, Hitachi) after 12 h. All of the permeation tests were carried out at least three times. The rejection (*R*) of each molecule was calculated from the following equation:

1 $$R = \left({\mathrm{1 - }}\frac{{C_{\mathrm {P}} \times V_2}}{{C_{\mathrm {f}} \times V_1}}\right){\mathrm{ \times 100\% }}$$

where *C*_f_ and *V*_1_ are the initial concentration and the volume of the feed solution on the surface of the membrane, respectively. *C*_p_ and *V*_2_ are the solute concentration in the permeate and the volume of the permeate in the container, respectively.

The rejection data for the membrane with PEGs were fitted to a log-normal pore size distribution. The pore size distribution of the cross-linked PTL membrane can be expressed by the following probability density function:^31^

2 $$\frac{{{\mathrm{d}}R\left( {d_{\mathrm{p}}} \right)}}{{{\mathrm{d}}d_{\mathrm{p}}}} = \frac{1}{{d_{\mathrm{p}}{\mathrm{In}}\sigma _{\mathrm{p}}\sqrt {{\mathrm{2\pi }}} }}{\mathrm{exp}}\left[ { - \frac{{\left( {{\mathrm{In}}d_p - {\mathrm{In}}d\mu _p} \right)^2}}{{{\mathrm{2}}\left( {{\mathrm{In}}\sigma _{\mathrm{p}}} \right)^2}}} \right]$$

where *d*_p_ is the pore size, *μ*_p_ is the mean pore size and *σ*_p_ is the geometric standard deviation.

The transport rate of molecules was calculated from the experimental data using the following equation:

3 $$J{\mathrm{ = }}\frac{{VC_{\mathrm{p}}}}{{tA}}{\mathrm{ \times 10}}^6$$

where *J* is the transport rate (nmol cm^−2^ h^−1^), *V* is the volume of the permeation solution (mL), *C*_P_ is the molecule concentration (mol L^−1^) in the permeation solution, *t* is the penetration time (h), and *A* is the effective area of the membrane (0.28 cm^2^).

### Dialysis experiments

The dialysis test cell had an effective area of 3 cm^2^ and was divided into two separated compartments. For the removal of middle-molecular-weight toxin, a mixture of solutes containing urea (1.5 mg mL^−1^), creatinine (0.1 mg mL^−1^), lysozyme (0.04 mg mL^−1^) and BSA (1 mg mL^−1^) was used as the feed solution. For the removal of indoxyl sulfate, a feed solution containing indoxyl sulfate (IS, 25 mg L^−1^) and BSA-FITC (1 mg mL^−1^) was used. The feed solution and dialysate were circulated at ambient temperature and a 10 mL min^−1^ flow rate, and 1 mL samples were collected from the feed solution at every 1 h interval for a total duration of 4 h. The clearance was calculated by Clearance (%) = (*C*_0_ − *C*_t_)/*C*_0_, where *C*_0_ and *C*_t_ are the concentrations of the target solute in the feed solution at time *t* = 0 and *t* = 2, 4, 6, and 12 h, respectively.

### Pressure-driven filtration for the separation of mixed dyes and proteins

A mixed solution of dyes (5 mL, 25 mg L^−1^ MO and MB) was filtered over the PTL/PET membrane at a constant pressure of 100 kPa. The concentration of both molecules was monitored simultaneously via UV-vis spectroscopy. The separation of proteins (5 mL, 25 mg L^−1^ BSA-FITC and insulin) followed the same process. The other pair of proteins consisted of MYO and insulin.

The surface porosity was calculated by Hagen-Poiseuille equation:

4 $$J{\mathrm{ = }}\frac{{{\it{\varepsilon }}{\mathrm{\pi }}r^2{\mathrm{\Delta }}{\it{p}}}}{{{\mathrm{8}}{\it{\mu L}}}}$$

the surface porosity *ε* is the area of pores against effective membrane surface, the pore radius is *r*, the pressure drop is Δ*p*, *μ* is water viscosity (8.9 × 10^−4^ Pa·s) and *L* is assumed to be equal to the thickness of the protein membrane.

### Preparation and size-selective separation of Au NPs by pressure-driven filtration

Two differently sized gold nanoparticles were prepared according to previously reported procedures with slight modifications. Briefly, citrate ion-coated gold nanoparticles (citrate-Au NPs) were prepared by reduction of a 20 mL aqueous solution containing 0.25 mM HAuCl_4_ and 0.25 mM trisodium citrate by the quick addition of 0.6 mL of a 0.1 M NaBH_4_ solution with stirring in ice-cold conditions^61^. Glutathione-coated gold nanoparticles (Glu-Au NPs) were synthesized and modified using the method of Zhou et al^62^. An aqueous solution of HAuCl_4_ (20 mL, 5 mM) was added into an aqueous glutathione solution (20 mL, 5 mM) under vigorous stirring at 90 °C for 35 min. The solution was centrifuged at 10,000 g for 5 min to remove the insoluble aggregates as well as large NPs. The mixture of gold nanoparticles containing 2 mL of citrate-Au NPs and 2 mL of Glu-Au NPs was then filtered under 1 bar of applied pressure using a vacuum filtration device (membrane diameter of 18 mm).

## Supplementary information

Supplementary Information

## Data Availability

All data supporting this study and its findings are available within the article and its Supplementary Information or from the corresponding author upon reasonable request.
