# Supplementary material for: Self-assembled membrane composed of amyloid-like proteins for efficient size-selective molecular separation and dialysis
Source: Nat Commun. 2018 Dec 21;9:5443. doi: 10.1038/s41467-018-07888-2 (PMC6303310; doi:10.1038/s41467-018-07888-2)
Supplement: Supplementary file 1 — Supplementary Information [file 41467_2018_7888_MOESM1_ESM.pdf]

Supplementary Information

Self-assembled membrane composed of amyloid-like proteins for efficient size-selective molecular separation and dialysis

Facui Yang, Fei Tao, Chen Li, Lingxiang Gao, Peng Yang

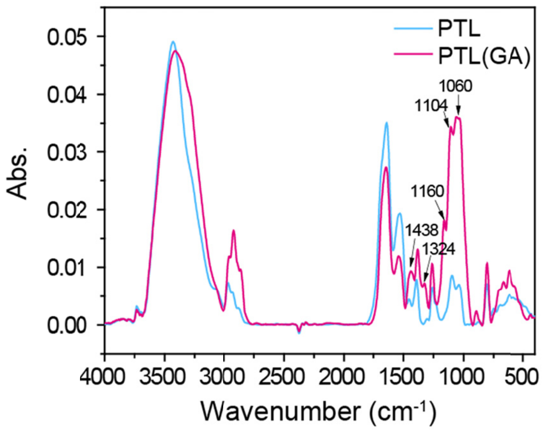

Supplementary Figure 1 | The FTIR spectra of the membranes before and after the crosslinking with glutaraldehyde (GA).

Supplementary Discussion: All the spectra showed strong amide II and I features in the 1500-1580 cm^{-1} (N-H bending and C-N stretching modes) and 1600-1700 cm^{-1} region (C=O stretching mode), respectively, and additionally exhibited pronounced signals at 1100-1300 cm^{-1} (C-N stretching and N-H bending modes). Clearly, the peak strength of amide I and II were reduced because amino amount decreased due to its conjugation with GA, and the peak height increases at 1160, 1000-1100 cm^{-1} (C-O-C) were observed in the GA-treated PTL. There were also new bands at 1438, 1324 cm^{-1} arising from CH_2 wagging observed in the GA-treated PTL. GA can react with several functional groups of proteins, such as amine, thiol, phenol, and imidazole because most reactive amino acid side-chains are nucleophiles, which could effectively react with GA. Among them, the predominant reaction was occurred between the aldehyde group (GA) and the amino group of the proteins to form Schiff base[1]. The mechanical properties of the membrane were improved by the intramolecular/intermolecular crosslinking via GA.

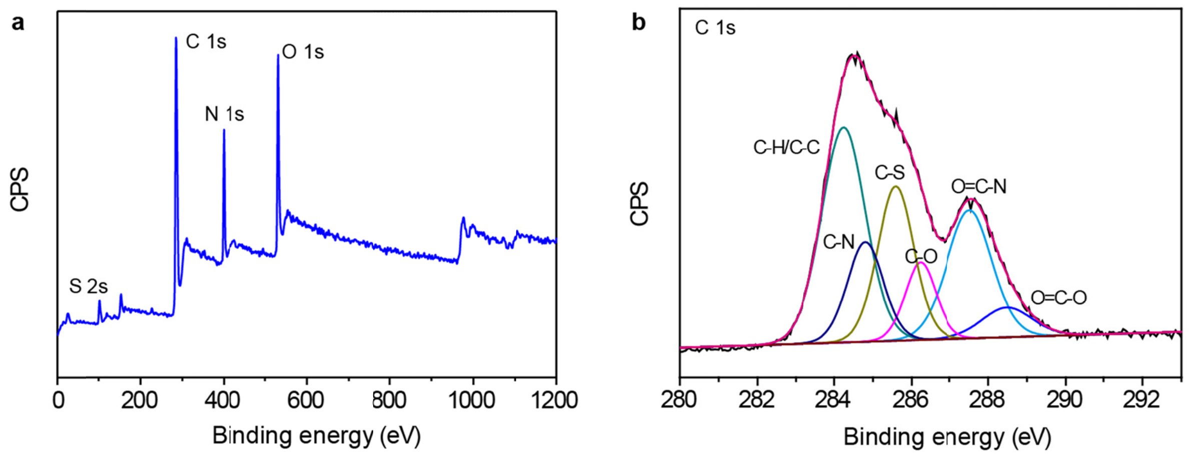

Supplementary Figure 2 | (a) XPS characterization of the cross-linked membrane. **(b)** High-resolution C 1s spectra of the cross-linked membrane. The deconvolution of C1s peak indicated that the membrane surface presented multiple functional groups mainly including alphatic carbon (C-H/C-C), amines (C-N), hydroxyls (C-O), thiols (C-S), amides (O=C-N) and carboxyl groups (O=C-O).

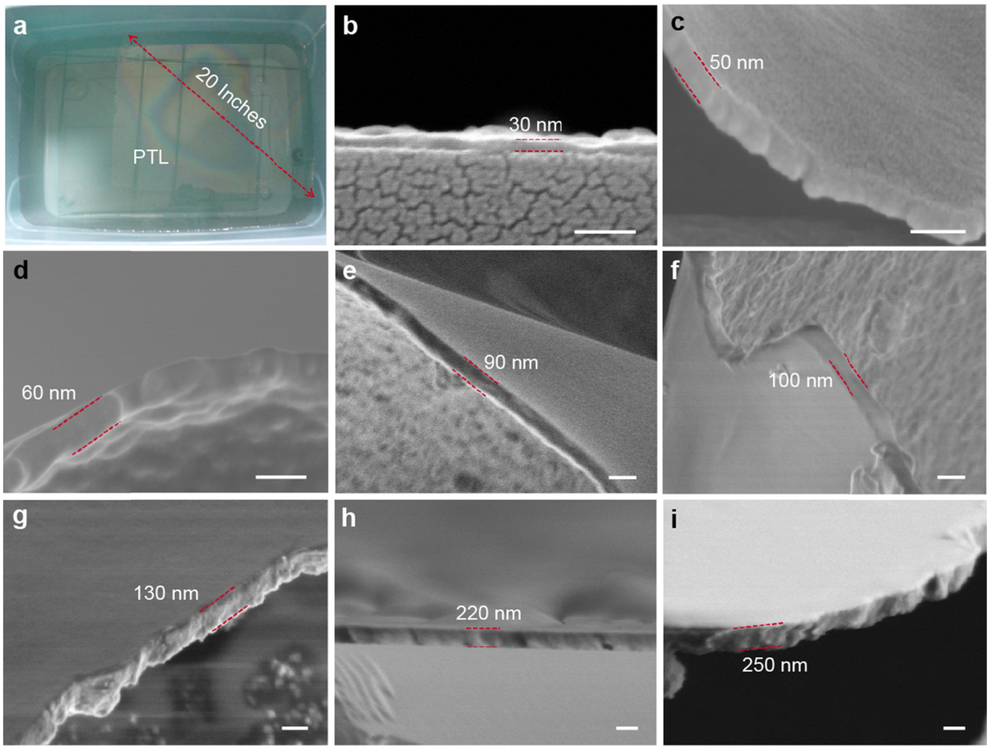

Supplementary Figure 3 | The macroscopic PTL membrane and its cross-sectional SEM image. (a) Photographic image of the PTL membrane with 20 in. in diagonal. **(b-i)** SEM images to show the cross-linked PTL membrane with lysozyme concentration at 1, 2, 4, 6, 8, 10, 20, 30 mg mL^{-1}, respectively. For other given conditions: 50 mM of TCEP, pH 7.0 and incubation for 120 min. Scale bars: **(b-d)** 100 nm, **(e-i)** 200 nm.

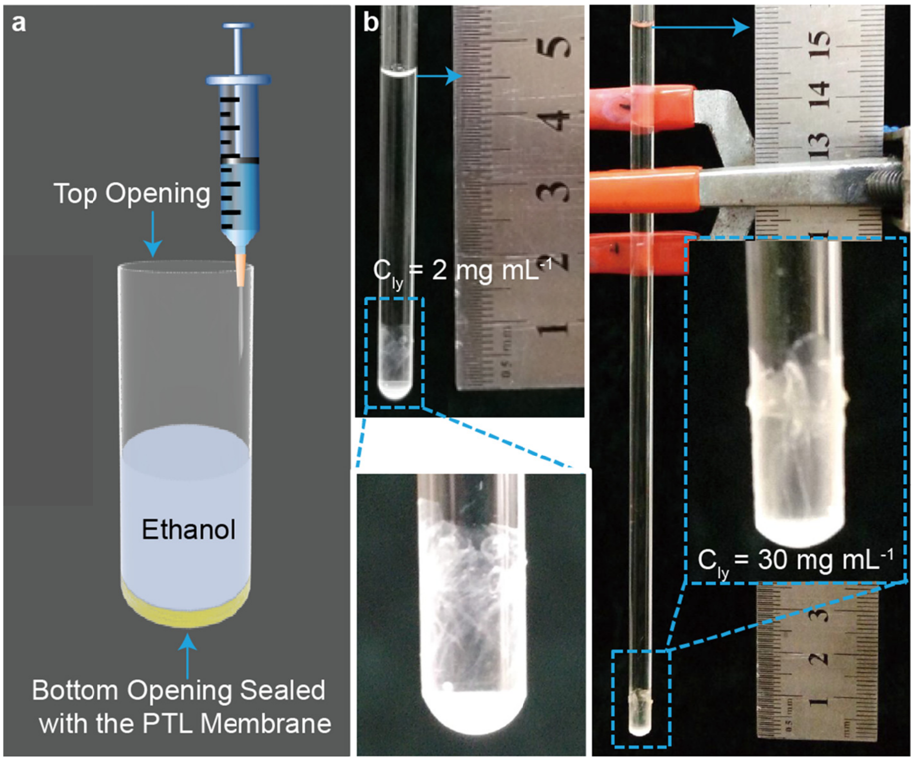

Supplementary Figure 4 | (a) Schematic representation of the setup to evaluate the pressure that the membrane could sustain. The tube was positioned vertically with the bottom opening being sealed with the PTL membrane. Ethanol was slowly introduced from the upper side to fill into the tube. **(b)** The photograph of ethanol column supported by the cross-linked PTL membrane (5 mm in diameter) with 50 (2 mg mL^{-1}) and 250 nm (30 mg mL^{-1}) in thickness. 50 and 250-nm-thick membranes could support the ethanol column of 4.5 cm (0.3 kPa) and 15.4 cm (1.2 kPa), respectively[2, 3].

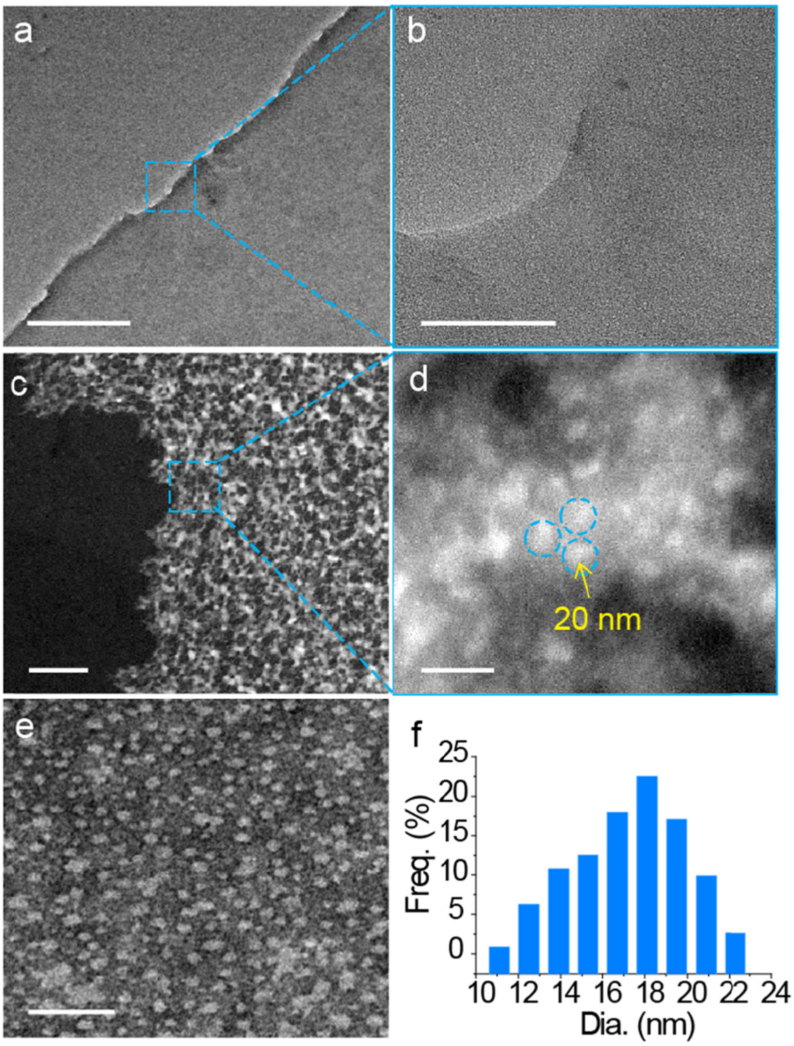

Supplementary Figure 5 | The TEM images of the PTL membrane: **(a)** TEM bright field image of a free-standing ultrathin membrane on a copper mesh, indicating the relatively smooth surface of the membrane. **(b)** The magnified TEM image of a square area in **(a). (c)** TEM dark field image of the membrane and **(d)** the corresponding high-resolution image in which three oligomers with a diameter of 20 nm were indicated. **(e)** TEM image of the resultant negatively stained membrane. **(f)** The corresponding size distribution of the oligomers in panel **(e)**. TEM images of the membranes at different scales with scale bars corresponding to **a-c** 500 nm, **d** 50 nm, **f** 100 nm.

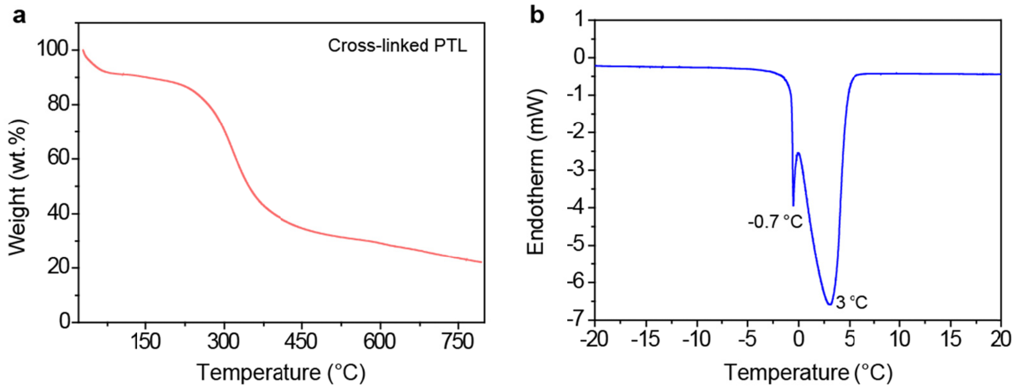

Supplementary Figure 6 | (a) The TG curve of the dried cross-linked PTL membrane. **(b)** The DSC thermogram of the hydrated cross-linked PTL membrane.

Supplementary Methods: For DSC measurement[4], the membrane with an area of 24 mm×24 mm was immersed in deionized water at room temperature for 12 h. The membrane was then saturated with water until no further weight gain was observed. The liquid water on the surface of wetted membranes was removed using a filter paper before weighing. About 2.2 mg of the wet saturated sample was then measured with DSC. The sample was first cooled to -30 °C and then heated to 30 °C at the rate of 2 °C min^{-1}. The heating process was monitored.

Supplementary Discussion: In the TG measurement, a mass loss (total 10 wt%) due to moisture evaporation of the internal water (adsorbed water) was observed from room temperature until 100 °C (boiling point of water at normal pressure). And after 100 °C, the decomposition then intensified above 200 °C to show a notable weight loss due to the decomposition of organic components, followed by a residue (20 wt%) at 800 °C. This study thus also showed that the membrane had a relatively high thermal-degradation temperature as 200 °C.

The corresponding results of DSC measurement showed the broad endotherm peak at 3 °C assigned to the melting of freezable free water on the wetted membrane. Regarding to the measurement of filled water (adsorbed water) in the pores of the membrane, it was reasonable to study the small but sharp endothermic peak found at -0.7 °C in the DSC curve, which was assigned to the melting of freezable surface-bound water in the pores of the membrane. Such melting temperature (-0.7 °C) was lower than the normal fusion temperature (0 °C) of ice, and this phenomenon was because that the freezing process of the surface-bound water competed with the water-material surface interaction[4].

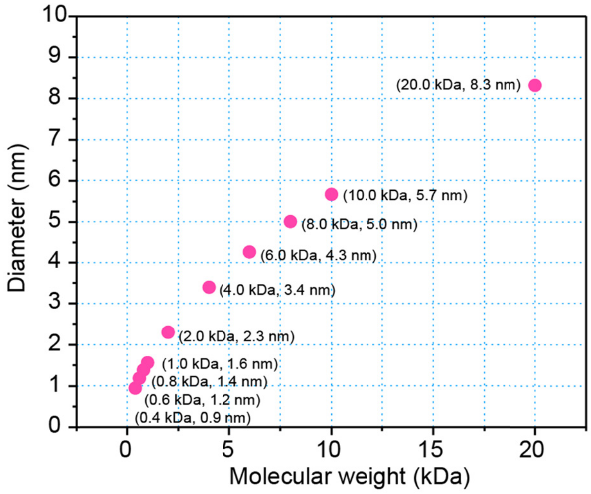

Supplementary Figure 7 | Stokes radii (r_s in cm) of PEG molecules as obtained from their molecular weights (M): $r_s = 16.73 \times 10^{-10} M^{0.557}$.

Supplementary Discussion: Firstly, the Stokes radius can be used to represent the effective solute size and is derived by assuming that the solute is spherical[5]. In our case, although PEG is a linear polymer from its molecular structure, it would be energetically favorable to adopt a configuration of random coil when it is dissolved in water under a dilute regime[6-8]. It has been reported to calculate the Stokes radius of PEG based on its random-coiling model[5]. Secondly, the use of Stokes radius requires that the dependence of solute separation on the steric and hydrodynamic interaction between the solute and the pore is negligible[5]. In our case, the separation may not be affected largely by the steric and hydrodynamic interaction between PEG and the membrane, because PEG is a neutral and flexible main-chain polymer without large side groups, which normally results in weak steric effect and interaction between the PEG and positively charged membrane surface. This condition was then supported by the evidences that the adsorption of PEG on the membrane was low and did not influence the separation performance of the membrane (**Supplementary Figure 8**). Thirdly, the Stokes radius is associated with diffusivity, and the corresponding calculation takes the assumption that diffusion is occurring inwardly through a cylindrical or slit-shaped pores[9]. In our system, the tortuous channels in the PTL membrane formed by close packing of spherical oligomers may fulfill this condition (**Figure 1g**), and the diffusion of PEG was caused by the concentration difference on both sides of the membrane with neglecting the deformation of PEG under osmotic pressure.

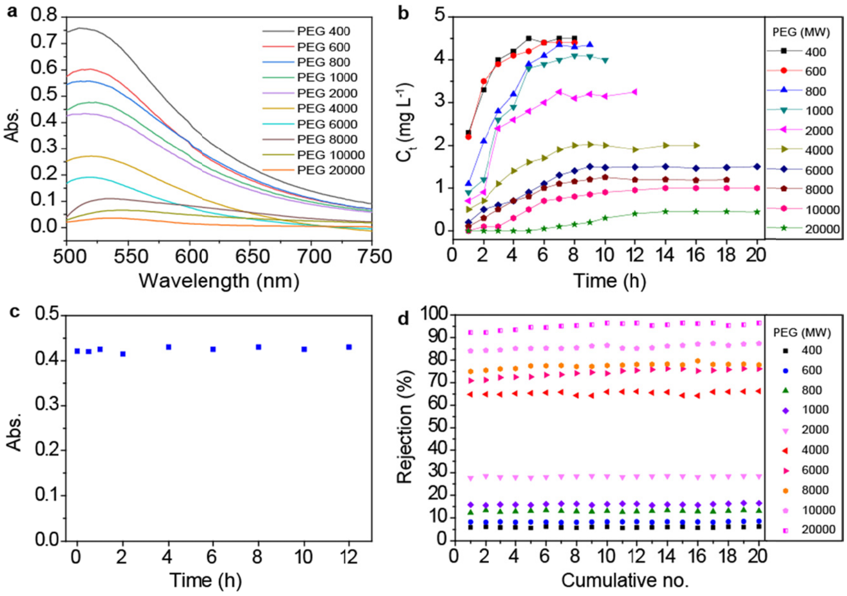

Supplementary Figure 8 | (a) UV-vis spectra to monitor the concentration of PEG molecules in the permeated solution to evaluate the mean pore size, size distribution and molecular weight cutoff (MWCO) of the PTL membrane (50 nm in thickness). For this aim, a certain amount of permeated PEG solution was diluted and added by 0.5 mL $BaCl_2$ solution (5%) and 0.5 mL I_2 solution (0.05 M), respectively. The absorbance was then measured at 520 nm after standing the mixed solution for 2 min[10]. **(b)** The diffusion of PEG with different molecular weight (0.5 g L^{-1} in the feed solution) through the membrane as a function of time, C_t, the concentration of PEG in the permeated solution at a given time. **(c)** The UV-vis absorption of PEG 2000 solution (0.5 g L^{-1}) after the solution contacting with the PTL membrane for different time. In this process, the PTL membrane supported by a PET substrate with a hole (dia. 6 mm) was entirely immersed in the PEG 2000 solution for a given time (no filtration was applied in this process), and then the UV-vis absorption of PEG solution at 520 nm after taking out the immersed membrane was recorded at different time. The results showed that the adsorption of PEG on the membrane was low, since the UV-vis absorbance of PEG solution (representing the concentration of PEG) after taking out the immersed membrane at different time did not change obviously from the beginning to at least 12 h. **(d)** Rejection of PEG with different molecular weight by the 50 nm-thick membranes for 20 times operation.

Supplementary Methods: The penetration experiments of PEG were performed at room temperature by using PEG-water mixtures (PEG, 500 mg L^{-1}) with different molecular weights. 50 μL PEG-water mixtures were added to the surface of the membrane that was pre-wetted by water. Filtrate was analyzed for their PEG concentrations by the UV-Vis spectrophotometer in order to

obtain the molecular weight distribution curve[10]. The rejection ratio was determined as a function of the molecular weight and the MWCO could be obtained from this curve. The rejection ratio was determined from the following equation:

$$R = (1-\frac{C_P \times V_2}{C_f \times V_1}) \times 100\% \quad (1)$$

Where, C_f and V_1 are the initial concentrations and the volume of the solution on the surface of the membrane. C_p and V_2 are the solute concentrations in the permeate and the volume of the permeate in the container, respectively.

MWCO was found at R = 90%. R was then plotted against the solute diameter d_p on a log-normal probability plot and the mean effective pore size μ_p can be found at R = 50%, and the geometric standard deviation σ_p was calculated from the ratio of d_p at R = 84.13% and 50%. The pore size distribution can be expressed by the probability density function:

$$\frac{dR(d_p)}{dd_p} = \frac{1}{d_p \ln\sigma_p \sqrt{2\pi}} \exp[-\frac{(\ln d_p - \ln d\mu_p)^2}{2(\ln\sigma_p)^2}] \quad (2)$$

Where d_p is the pore size, μ_p is the mean pore size and σ_p is the geometric standard deviation.

Supplementary Discussion: The PEG solutions used herein had the highest concentration of 0.05% (w/v), which is well below the overlap concentration (30%) for PEG in the semidilute regime. In the semidilute regime and when the radius of the chain is larger than the pore diameter, the molecules are expected to enter the pore only if the polymer concentration is sufficiently large. It shows that the chains are extracted from the semidilute solution and then contact with a pore by a reptation mechanism[11, 12]. In this context, since the concentration of PEG used in the present work was in a dilute regime in which the polymer chains were not entangled, the reptation mechanism may not be suitable for explaining our results. Alternatively, Bezrukov has carried out the experiments concerning the transport of PEG in a dilute regime[13]. In this regime, if the polymers are too large to be accommodated within the pore, the out-of-the-pore part of the molecule pulls on the trapped part, thus acting as an entropic spring. On the one hand, water-soluble polymers with characteristic sizes being much smaller than pore diameters, would easily enter into the pores; on the other hand, large polymers are excluded from pores because of entropic or steric reasons (e.g. for polymers comprising bulky monomers). Similarly, in our system, small PEG chains could easily pass through the PTL membrane, and the rejection ratio elevated as the molecular weight (i.e. the chain size) increased.

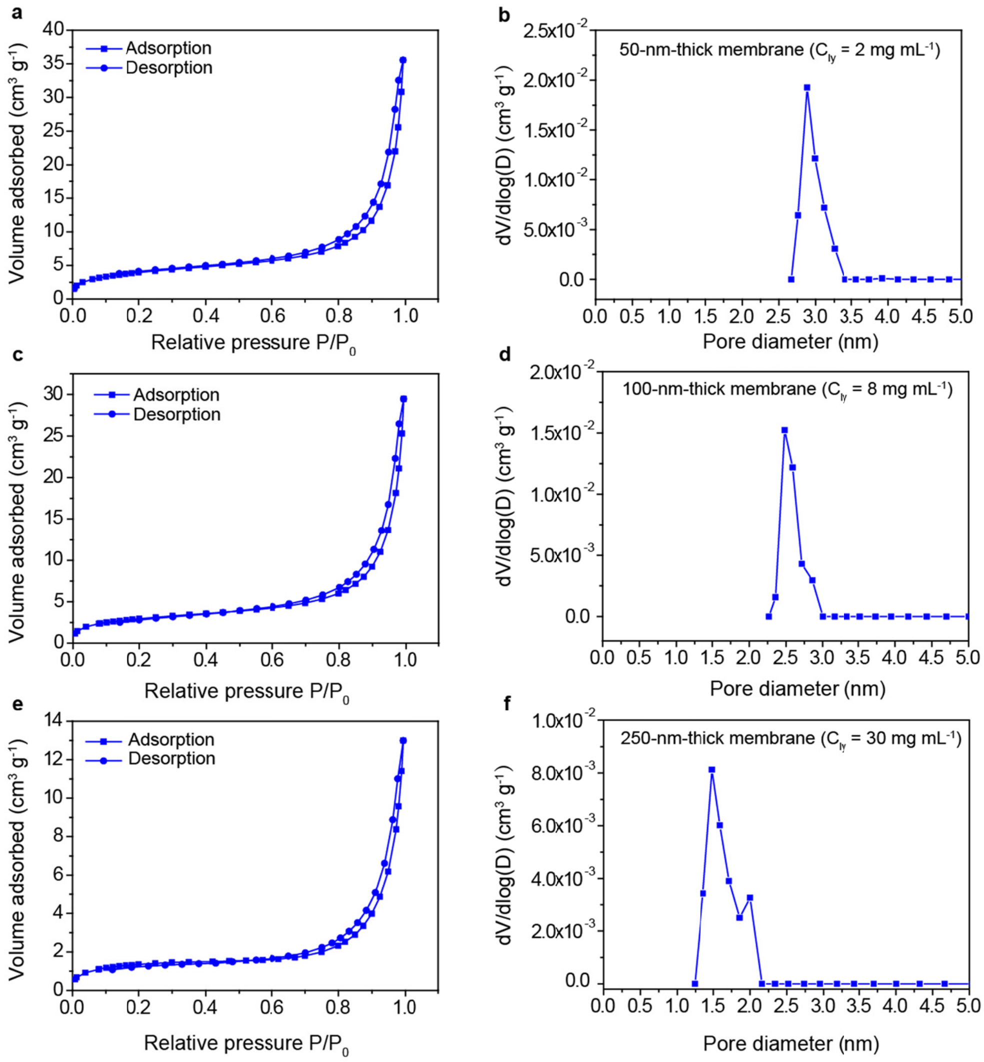

Supplementary Figure 9 | Nitrogen adsorption-desorption isotherms and corresponding pore size distributions of 50-nm-thick membrane (**a**, **b**), 100-nm-thick membrane (**c**, **d**) and 250-nm-thick membrane (**e**, **f**).

Supplementary Discussion: The curves (**a**, **c**, **e**) had the characteristic feature of the type IV isotherm and hysteresis loop. The rapid nitrogen uptake ($P/P_0 > 0.9$) might be due to large voids formed by an accumulation of the multiple membranes. The BJH adsorption cumulative volume of pores and BET specific surface area of the membranes with different thickness were further derived from the measurements around 0.05 cm^3 g^{-1} and 14.2 m^2 g^{-1} (50-nm-thick), 0.04 cm^3 g^{-1} and 10.6 m^2 g^{-1} (100-nm-thick), and 0.02 cm^3 g^{-1} and 4.7 m^2 g^{-1} (250-nm-thick), respectively.

The data of BET surface area (4.7 m^2 g^{-1}) and pore volume of the 250-nm-thick membrane (0.02 cm^3 g^{-1}) were higher than those (BET surface area 1.3 m^2 g^{-1} and pore volume 0.006 cm^3 g^{-1}) of semipermeable eggshell membrane with selective permeability[14].

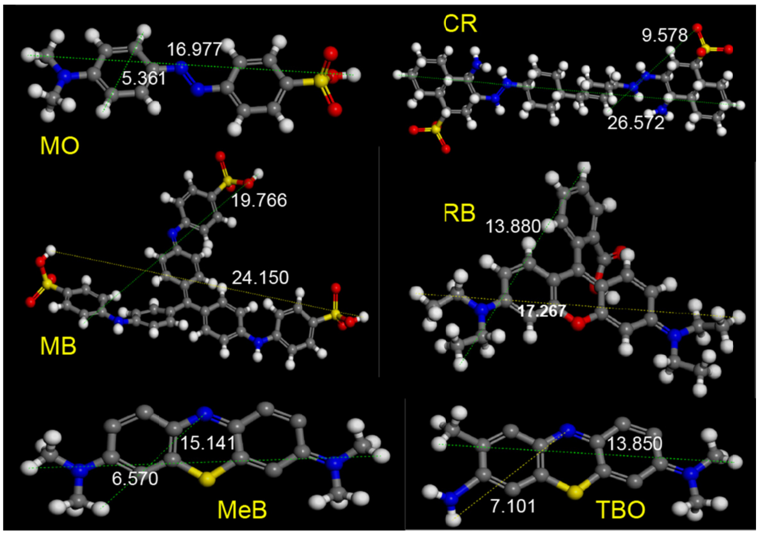

Supplementary Figure 10 | The molecular structures of methyl orange (MO), Congo red (CR), methyl blue (MB), rhodamine B (RB), methylene blue (MeB), and toluidine blue O (TBO) depicted using Materials studio 7.0. The unit of the molecular size shown in the image is angstrom (Å).

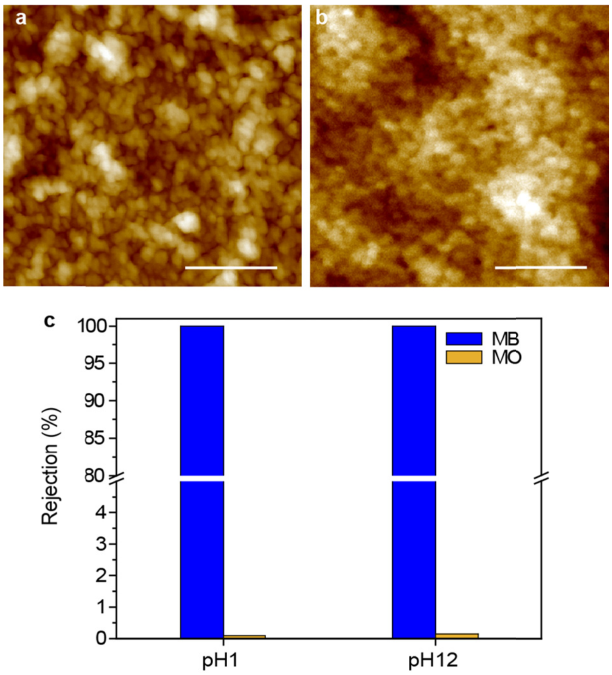

Supplementary Figure 11 | The effect of pH on the membrane stability. AFM images of the membrane after treating with acidic (pH 1) **(a)** and alkaline (pH 12) aqueous solution **(b)**. The membranes are soaked in acidic and alkaline aqueous solution for 12 h, respectively. **(c)** Separation of mixed dyes (MB and MO) by the membrane after treating with acidic (pH 1) or alkaline (pH 12) aqueous solution. AFM images of the membranes at same scales with 120 nm.

Supplementary Methods: In this figure, the stability test of the cross-linked PTL membrane in acidic (pH 1) or alkaline (pH 12) aqueous solution was studied. The membranes were soaked in acidic (pH 1) or alkaline (pH 12) aqueous solution for 12 h, and after that, the surface morphology and separation performance of the membranes were evaluated. It turned out by atomic force microscopy (AFM) that the surface morphology of the membrane barely changed in acidic (pH 1) or alkaline (pH 12) aqueous solution. For the separation test, the mixed dyes of MO and MB could be still separated effectively by the membrane after treating with acidic (pH 1) or alkaline (pH 12) aqueous solution, without detectable retardation on the separation performance. Strong basic pH may destroy the secondary structure of a native protein (e.g., β-sheet), and the above results indicated that the water-insoluble β-sheet-rich amyloid-like protein aggregates (the PTL membrane) showed an enhanced stability at the pH 1 or 12.

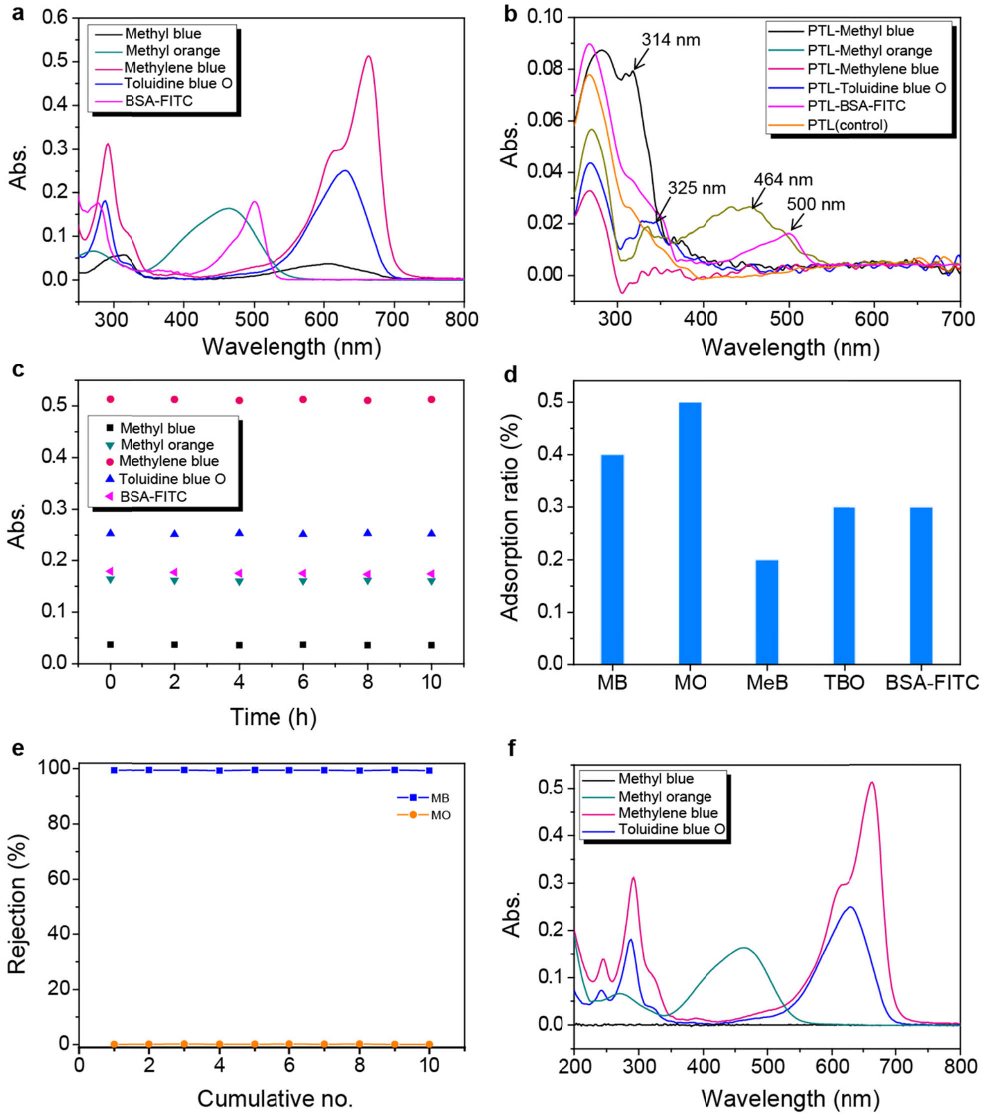

Supplementary Figure 12 | (a) The UV-vis spectra of the solutions of methyl blue (MB), methyl orange (MO), methylene blue (MeB), toluidine blue O (TBO) or BSA-FITC (250 mg L^{-1}, diluted 50 times for the test). **(b)** The UV-vis spectra of the membranes after adsorbing MB, MO, MeB, TBO or BSA-FITC for 12 h, respectively. **(c)** The UV-vis absorption (at determined wavelength) of the solutions for MB (at 604 nm), MO (at 464 nm), MeB (at 664 nm), TBO (at 630 nm) or BSA-FITC (at 500 nm) respectively after the solutions (5 mL, 5 mg L^{-1}) contacting with the PTL membrane for different time. **(d)** The adsorption ratios of dyes or proteins on the membranes after reaching adsorption equilibrium for MB, MO, MeB, TBO or BSA-FITC. **(e)** Separation of mixed dyes: 250 mg L^{-1} MB and 250 mg L^{-1} methyl orange (MO) by the same membrane for ten consecutive cycles. **(f)** The UV-vis spectra of the permeated solution after the diffusion of MB, MO, MeB or TBO through the membrane for 6 h (the initial concentration of all dyes was 250 mg L^{-1}). MO, TBO and

MeB could pass through the membrane, and the membrane effectively rejected MB.

Supplementary Methods: For the process in panel **a** and **b**, three membranes (24×24 mm) simply floated on the surfaces of the dye or protein solutions (250 mg L^{-1}) for 12 h, and after that, the membranes were taken out from the solution, rinsed by copious water and then subjected to the UV-vis measurement.

For panel **c**, the PTL membrane supported by a PET nuclear pore membrane was entirely immersed in the solutions for a given time (no filtration was applied in this process), and then the UV-vis absorption of the solutions at 604 nm (for MB), 464 nm (for MO), 664 nm (for MeB), 630 nm (for TBO) or 500 nm (for BSA-FITC) after taking out the immersed membrane was recorded at different time. The corresponding absorptions did not change obviously from the beginning to at least 10 h, indicating a low adsorption extent of dyes or proteins on the membrane.

For panel **d**, three membranes (24×24 mm) were entirely immersed in the dyes or BSA-FITC solutions (5 mg L^{-1}) for 12 h, the adsorption ratio (R) of the adsorbed dyes or BSA-FITC on the membrane were calculated from the concentration change in their aqueous solution before (C_0) and after (C_t) adsorption equilibrium, $R = (C_0\text{-}C_t)/C_0$.

Supplementary Discussion: The surface state of the amphiphilic proteinaceous PTL material could be affected by the aqueous microscopic environment. In such a case, the hydrophilic amino acid residues exposed on the PTL material surface supported a surface hydration effect after the PTL material was incubated in a biological media or buffer, and it was thus rationalized that such hydration potency nearby the surface might suppress the non-specific adsorption of species in the solution.

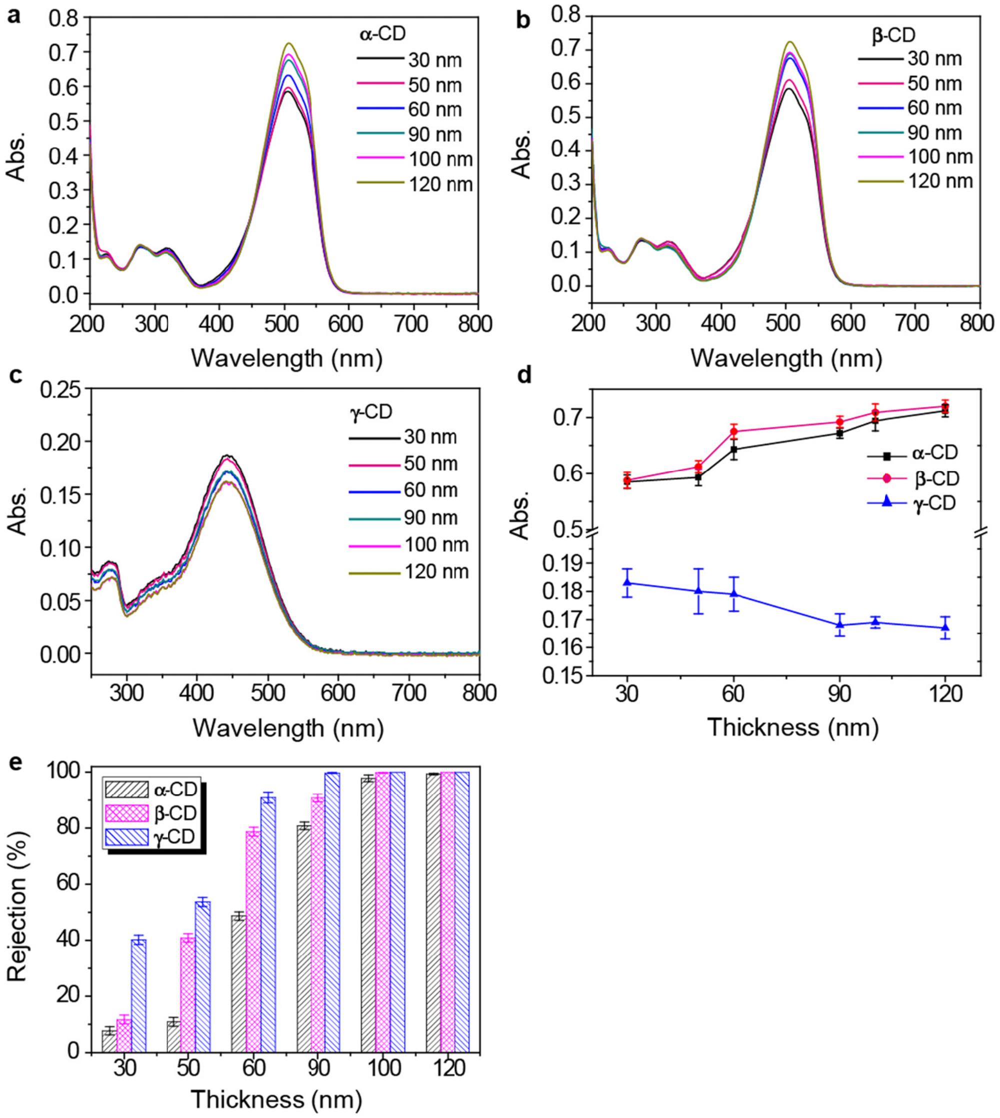

Supplementary Figure 13 | Penetration of cyclodextrin by the PTL membranes with different thickness. The formation of the inclusion complex of MO with α-CD **(a)**, β-CD **(b)**, and BCG with γ-CD **(c)** was monitored by UV-vis absorption spectroscopy. **(d)** The absorption of the inclusion complexes of MO (at 505 nm) with α-CD, β-CD**,** and BCG (at 445 nm) with γ-CD in the permeated solution as a function of the membrane thickness (i.e. the decrease of the pore size). **(e)** The effect of the membrane thickness on the separation of cyclodextrins (α, β, γ). Three repeated tests were performed for each set of measurements and errors are derived as the deviation from the average.

Supplementary Methods: The inclusion complex formation of methyl orange with α-CD (or β-CD) (as called MO method) and bromocresol green with γ-CD (as called BCG method) were monitored by UV absorption spectroscopy[15, 16]. For the determination of α-CD (or β-CD) concentrations in the filtrate by using MO method, 1 mL of α-CD (or β-CD) solution was mixed with 0.01 mL of 1 mg

mL^{-1} and 0.01 mL of 1 M HCl. Then the differential UV absorption spectrum was measured. For the determination of γ-CD concentrations in the filtrate by using BCG method, 1 mL of γ-CD solution was mixed with 0.01 mL of 5 mM BCG (in 20% ethanol solution) and 0.01 mL of 1 M HCl.

Supplementary Discussion: It was clear that the protein-surrounded channels in the PTL membrane distinguished the sizes of cyclodextrins. The addition of either α-CD or β-CD to an acidic solution of MO was found to decrease the absorbance at 505 nm (a characteristic peak for MO) (**Supplementary Fig. S13a, b**). The amounts of γ-CD in the samples were determined with the BCG method, and the absorbance at 445 nm (a characteristic peak for BCG) increased with increasing the concentration of γ-CD (**Supplementary Fig. S13c**).

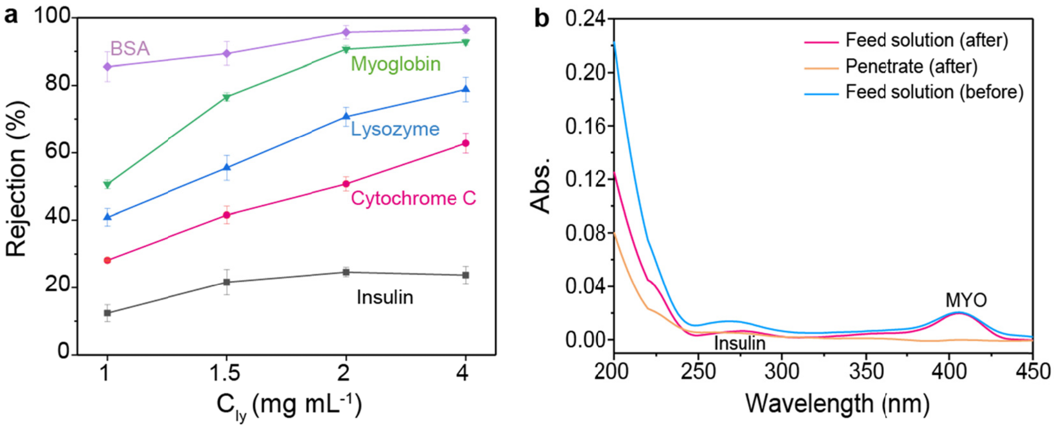

Supplementary Figure 14 | (a) The effect of the lysozyme concentration on the rejection of proteins by the PTL membrane. **(b)** UV/Vis spectra of the protein mixture (MYO, λ_{max} = 410 nm and insulin, λ_{max} = 280 nm) before and after undergoing the penetration. Data shown are the average of triplicates with standard deviation.

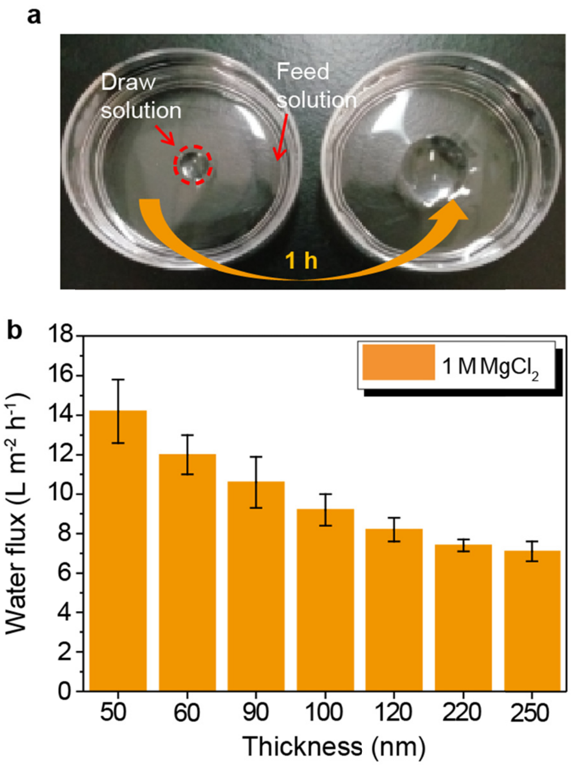

Supplementary Figure 15 | (a) The image showing that water transported across the cross-linked membrane due to the concentration difference between the two sides of the membrane. The original volume of liquid drop was 10 μL 1 M $MgCl_2$. **(b)** The water flux in the FO process as a function of the membrane thickness. The draw solution was 50 μL 1 M $MgCl_2$, and water was used as feed solution. Data shown are the average of triplicates with standard deviation. Note the assignment of draw solution and feed solution in this figure and **Supplementary Fig. 16** is slightly different from those in the setups in **Fig. 4c**, **Supplementary Fig. 17** and **Fig. 18**, in which the salt solution was termed feed solution and pure water side was denoted as dialysate side.

Measurement of water flux: The water flux (F_w, L m^{-2} h^{-1}) was calculated by the following equations: $F_W = Q/St$, where Q, S and t represent the volume of permeate (L), the effective membrane area (m^2) and the permeation time (h), respectively[17].

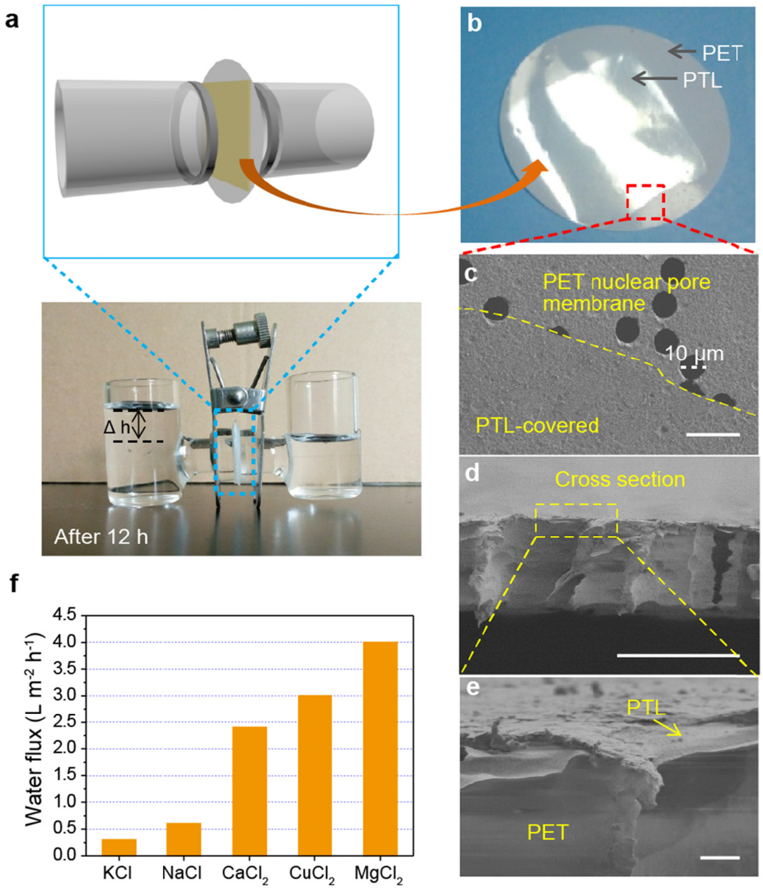

Supplementary Figure 16 | (a) Schematic representation of the setup for the water transport experiments. The liquid volume of each cell was 10.0 mL at the beginning of the experiments. **(b)** The optical photograph of the PTL/PET membrane. **(c)** The corresponding SEM image for the top surface of the PTL/PET nuclear pore membrane, showing the PTL layer on the PET surface (left lower side from the dotted boundary line) and uncovered PET surface (right upper side from the dotted boundary line). **(d)** The cross-sectional SEM image showing the thickness of PET in 12 μm and high-magnification image **(e)**. **(f)** The water flux as a function of electrolyte types. The draw solution was 0.1 M salt solution (left cell in **(a)**), and water was used as feed solution (right cell in **(a)**), and the liquid level difference between two cells after 12 h transport was Δh. Scale bars in **c**, **d** are 20 μm, in **e** is 2 μm.

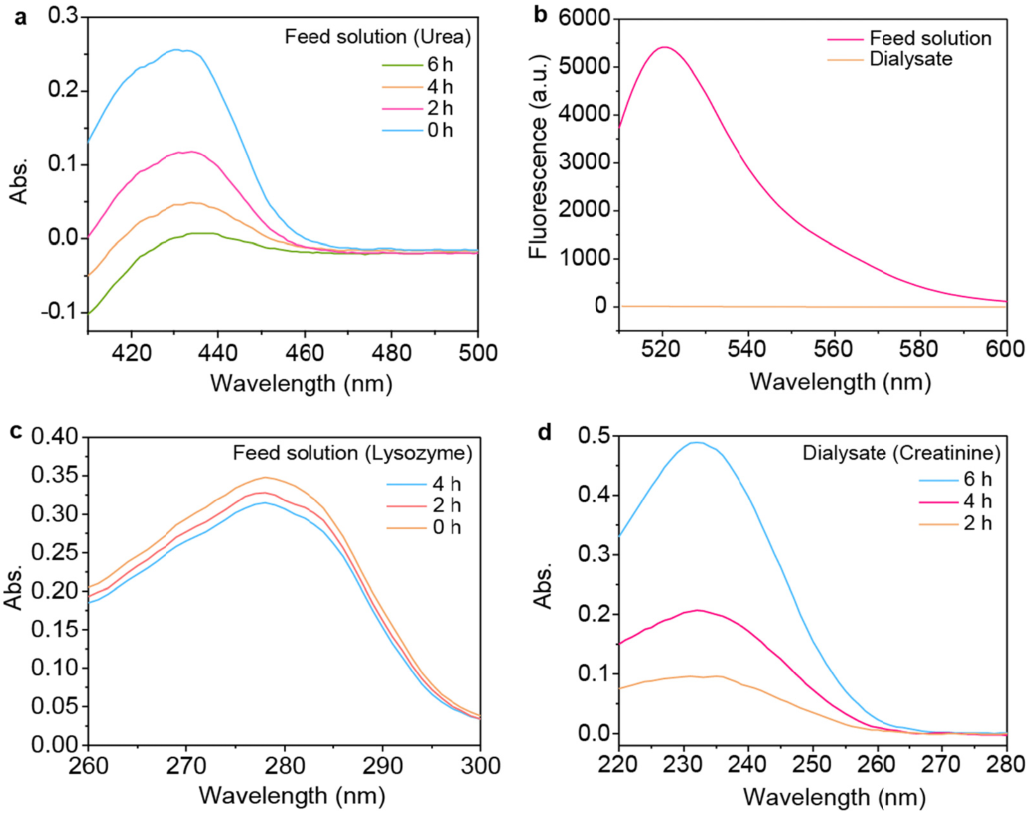

Supplementary Figure 17 | (a) UV-vis spectra measured on the feed side of the membrane for urea clearance at different hemodialysis (HD) time. **(b)** Fluorescence spectra of the feed solution and dialysate for BSA-FITC after 6 h dialysis (at 40 times dilution). **(c)** UV-vis spectra measured on the feed side of the membrane for lysozyme clearance at different HD time. **(d)** UV-Vis spectra of creatinine in the dialysate side at different HD time.

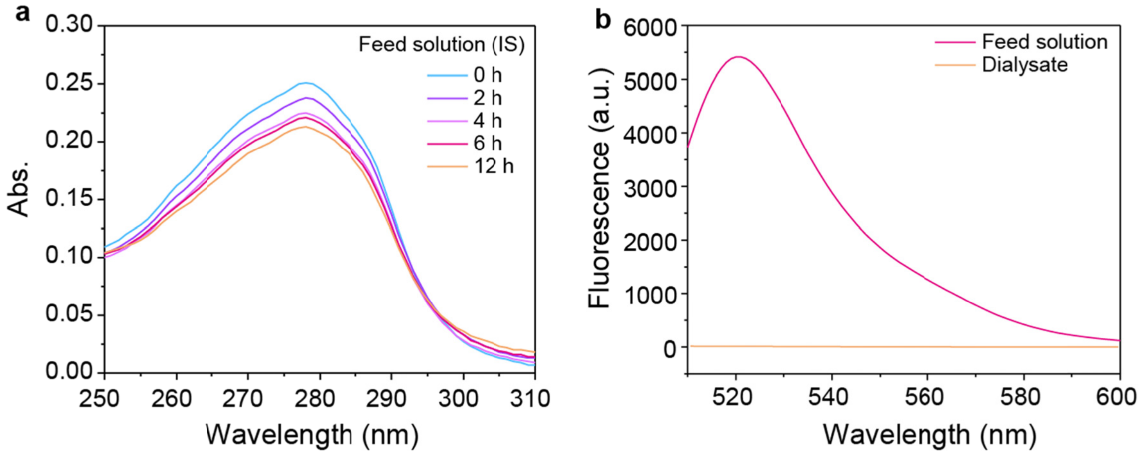

Supplementary Figure 18 | (a) UV-vis spectra measured on the feed side of the membrane for indoxyl sulfate (IS) at different HD time. **(b)** Fluorescence spectra of the feed solution and dialysate for BSA-FITC after 12 h dialysis (at 40 times dilution). In this experiment, 20 mL original mixture solution was used containing 1.0 mg mL^{-1} BSA and 25 mg L^{-1} indoxyl sulfate.

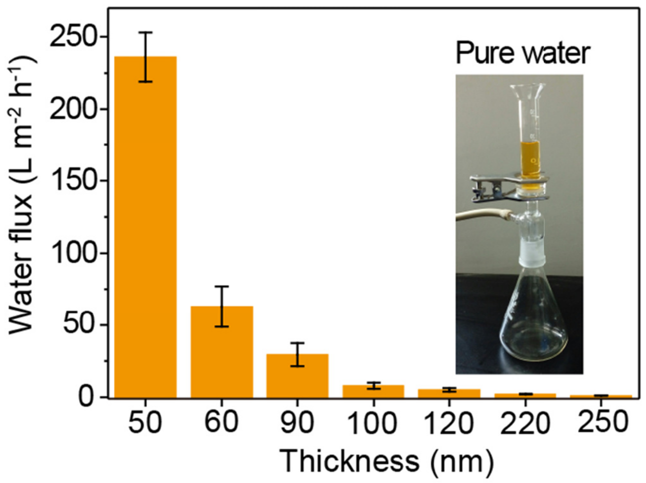

Supplementary Figure 19 | The thickness-dependent changes in permeability of pure water through the pressure-driven filtration on the PTL membrane, creating trans-membrane pressures up to 100 kPa. Optical photographs of the setup and the membrane for separation experiment (inset). Data shown are the average of triplicates with standard deviation.

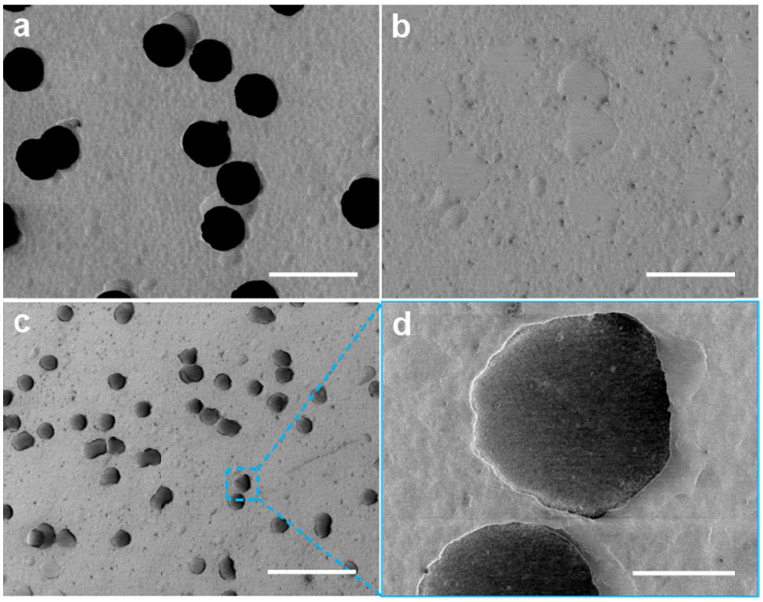

Supplementary Figure 20 | SEM images of **(a)** blank PET nuclear pore membrane and **(b)** the cross-linked PTL membrane covering on the PET nuclear pore support. **(c)** SEM image of the PTL/PET membrane after the pressure-driven filtration and **(d)** the magnified image showing no damage found in the cross-linked PTL membrane. In comparison with the PTL membrane covering laterally onto PET nuclear pore support before pressure-driven filtration (**Supplementary Fig. 16**), there was no damage observed on the PTL membrane covering laterally onto PET nuclear pore support after pressure-driven filtration, and the PTL membrane on the PET nuclear pore support after the pressure-driven filtration only showed certain bending deformation due to its flexibility responding to a pressure field. Scale bars: **a**, **b** 20 μm, **c** 50 μm, **d** 5 μm.

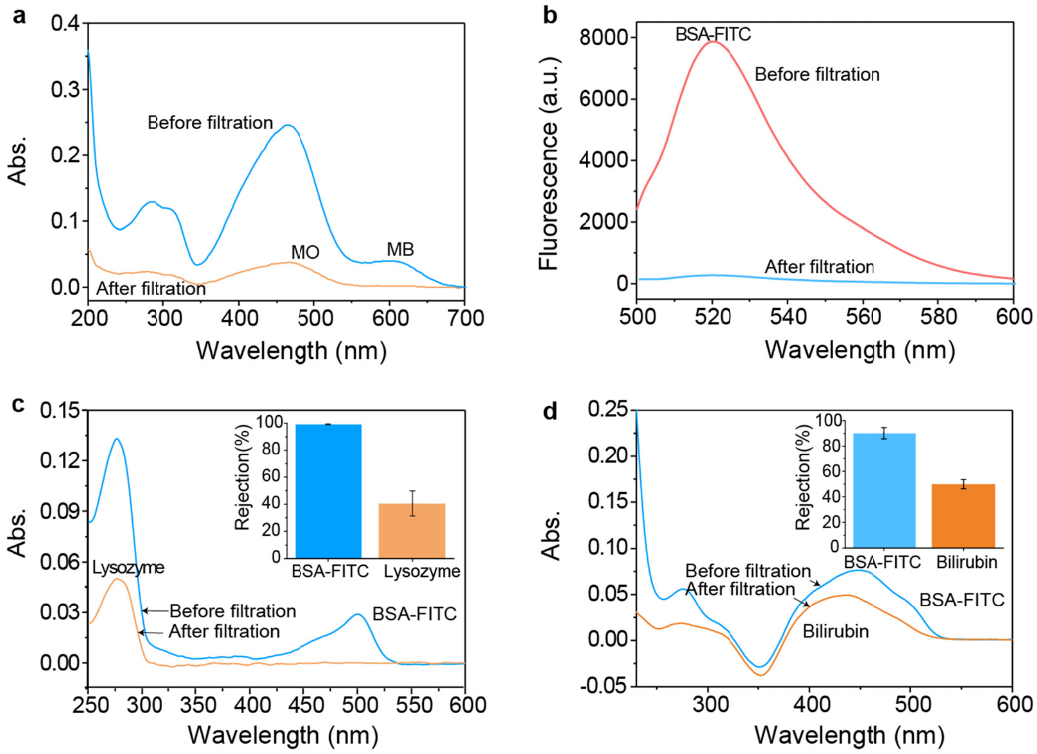

Supplementary Figure 21 | The pressure-driven filtration through the PTL membrane for dyes and proteins: (a) UV/Vis spectra of the dye mixture: MO (50 mg L^{-1}, λ_{max} = 464 nm) and MB (50 mg L^{-1}, λ_{max} = 609 nm) before and after the filtration (at 200 times dilution). **(b)** Fluorescent spectra of the protein mixture of BSA-FITC (25 mg L^{-1}, λ_{max} = 520 nm) and insulin before and after the filtration. **(c)** UV/Vis spectra of the protein mixture of BSA-FITC (25 mg L^{-1}, λ_{max} = 500 nm) and lysozyme (25 mg L^{-1}, λ_{max} = 280 nm) before and after the filtration and the inset as the rejection ratios of BSA-FITC and lysozyme from the mixture. **(d)** UV/Vis spectra of the mixture of BSA-FITC (25 mg L^{-1}, λ_{max} = 500 nm) and bilirubin (25 mg L^{-1}, λ_{max} = 430 nm) before and after the filtration and the inset as the rejection ratios of BSA-FITC and bilirubin from the mixture. Data shown are the average of triplicates with standard deviation.

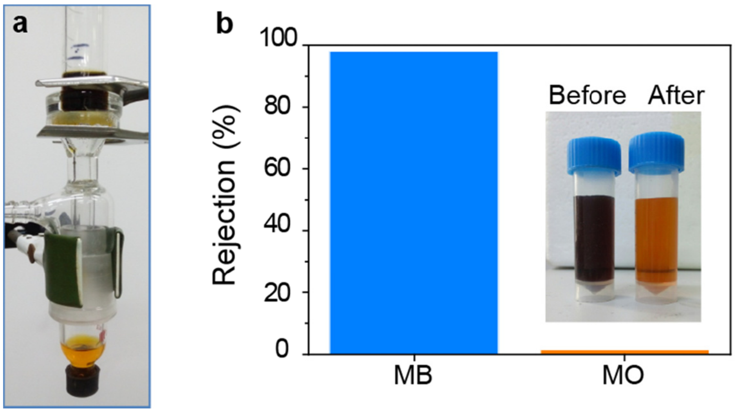

Supplementary Figure 22 | Separation stability evaluation of the cross-linked PTL membrane by ethanol treatment. **(a)** Optical photograph of the pressure-driven filtration setup to separate the mixed dyes (MB and MO). **(b)** The rejection of MB and MO passing through the ethanol-treated PTL membrane and the inset as the optical photograph of the mixed dye solution before and after the filtration.

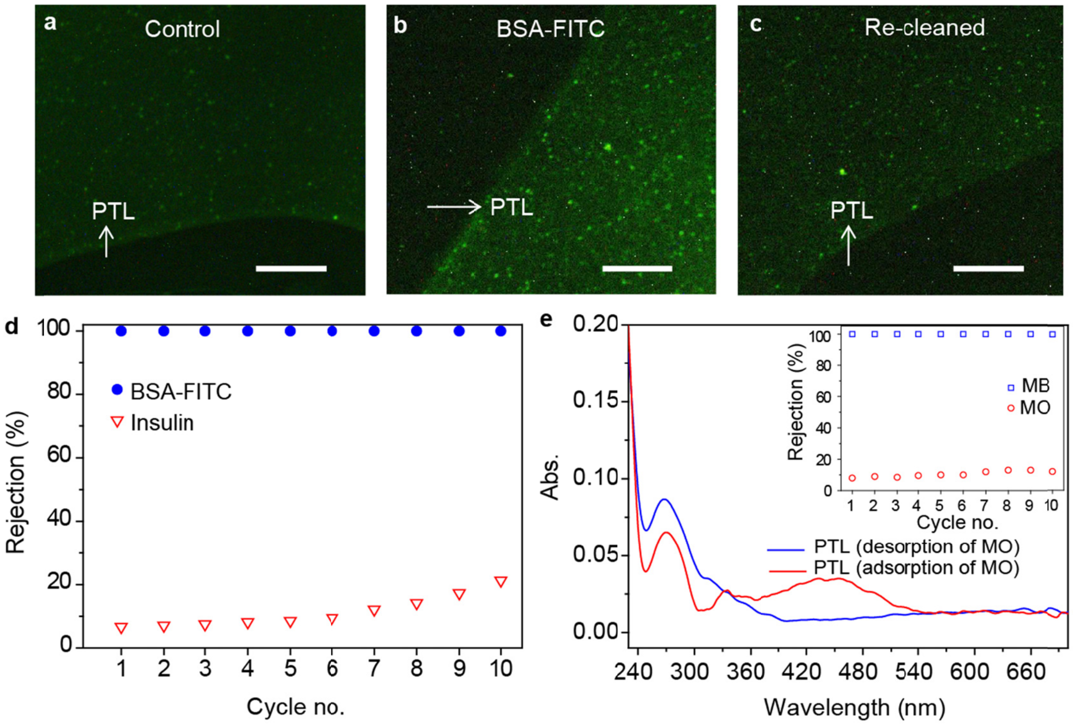

Supplementary Figure 23 | Fluorescence microscopy images of the membranes before **(a)** and after the adsorption of BSA-FITC **(b)**, and **(c)** the membrane re-cleaned (to remove adsorbed BSA-FITC) by using 3 M guanidine hydrochloride aqueous solution. **(d)** The rejection ratios of BSA-FITC and insulin from their mixture through the same regenerated membrane for 10 cycles by the pressure-driven filtration. **(e)** UV-vis spectra of the membranes after adsorbing and desorbing (regenerating in 0.01 M HCl for 4 h) MO, respectively. The inset figure was the rejection ratios of MB and MO from their mixture through the same regenerated membrane for 10 cycles by the pressure-driven filtration. Scale bars in **a**, **b** and **c** are 100 μm.

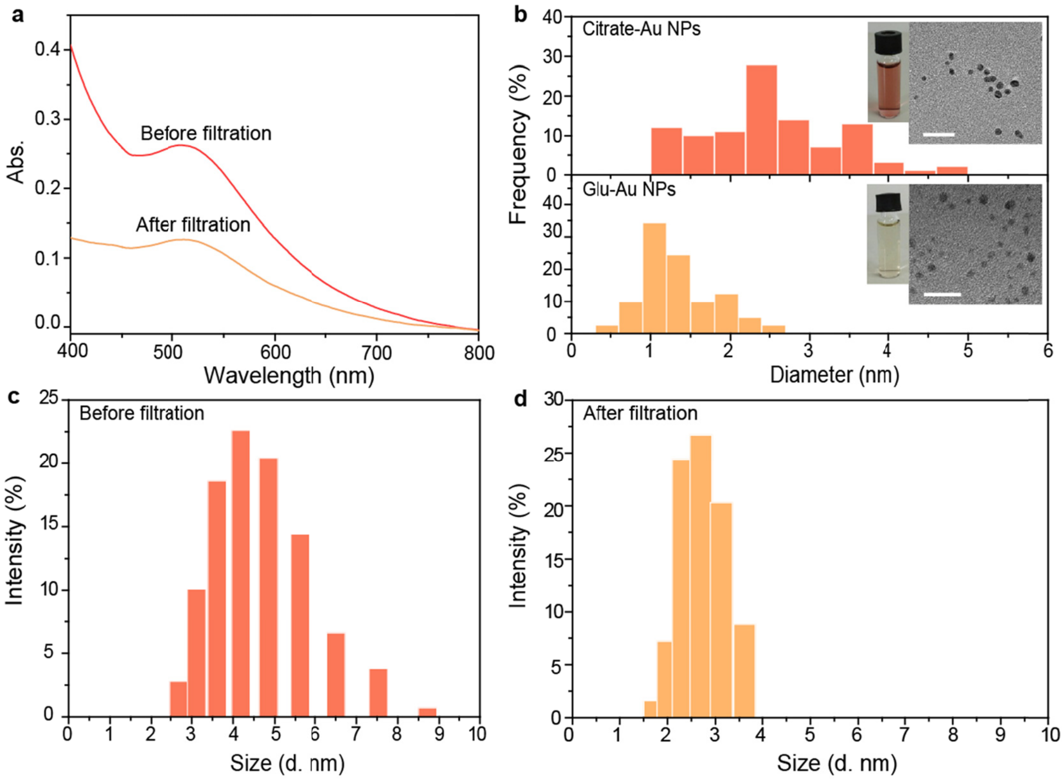

Supplementary Figure 24 | The pressure-driven filtration through the PTL membrane for gold nanoparticles: (a) UV/Vis spectra of the gold nanoparticles (NPs) mixture of Citrate-Au NPs (λ_{max} = 510 nm) and Glu-Au NPs (λ_{max} = 509 nm) before and after the filtration. **(b)** The size distribution curves, optical photographs and TEM images of gold nanoparticles for Citrate-Au NPs solution (top panel, diameter: 1-5 nm) and Glu-Au NPs solution (bottom panel, diameter: 0.5-2.5 nm). **(c and d)** DLS results showing the size distribution of the Au NPs in aqueous solution before **(c)** and after the filtration (in the filtrate) **(d)**. The inset TEM images of **b** with different scale bars: 20 nm (up), 2 μm (down).

Supplementary Table 1. Physical properties of the tested molecules as well as the corresponding rejection performance of 50-nm-thick PTL membranes.

Molecular	MW ($g\ mol^{-1}$)	Concentration ($g\ L^{-1}$)	Size (hydrated dia. nm)	Type	Rejection %
α-CD	972.846	2.0	1.9	Neutral	10.9 ± 1.90
β-CD	1134.98	2.0	2.0	Neutral	40.9 ± 1.50
γ-CD	1297.12	2.0	2.2	Neutral	53.7 ± 1.60
Methyl orange	327.334	0.5	1.2	Anionic	0.80 ± 0.10
Toluidine blue O	305.824	0.5	1.4	Cationic	1.20 ± 0.50
Methylene blue	319.851	0.5	1.2	Cationic	0.80 ± 0.20
Congo red	696.664	0.5	2.3	Anionic	12.1 ± 1.00
Rhodamine B	479.017	0.5	1.8	Cationic	1.40 ± 0.20
Methyl blue	799.795	0.5	3.0	Anionic	99.6 ± 0.10
Cytochrome C	884.893	10	3.0	Anionic	60.5 ± 2.10
Bovine serum albumin	66,463.0	10	7.2	Anionic	98.5 ± 0.10
Lysozyme	14,388.0	10	3.1	Cationic	66.6 ± 1.30
α-Lactalbumin	14,178.0	10	3.1	Anionic	98.1 ± 1.20
Insulin	5,807.69	10	2.4	Anionic	22.6 ± 3.80
Myoglobin	16,700.0	10	3.5	Neutral	90.6 ± 3.10

Supplementary References

1. Migneault, I., Dartiguenave, C., Bertrand, M. J., & Waldron, K. C. Glutaraldehyde: behavior in aqueous solution, reaction with proteins, and application to enzyme crosslinking. *Biotechniques* **37**, 790-796 (2004).
2. Peng, X., Jin, J., Nakamura, Y., Ohno, T. & Ichinose, I. Ultrafast permeation of water through protein-based membranes. *Nat. Nanotechnol.* **4**, 353-357 (2009).
3. Vendamme, R., Onoue, S. Y., Nakao, A., & Kunitake, T. Robust free-standing nanomembranes of organic/inorganic interpenetrating networks. *Nat. Mater.* **5**, 494-501 (2006).
4. Yu, S. K., Dong, L., Hickner, M. A., Glass, T. E., Vernon Webb, A., & Mcgrath, J. E. State of water in disulfonated poly(arylene ether sulfone) copolymers and a perfluorosulfonic acid copolymer (nafion) and its effect on physical and electrochemical properties. *Macromolecules* **36**, 6281-6285 (2003).
5. Singh, S., Khulbe, K. C., Matsuura, T. & Ramamurthy, P. Membrane characterization by solute transport and atomic force microscopy. *J. Membrane Sci.* **142**, 111-127 (1998).
6. Huang, L., & Nishinari, K. Interaction between poly(ethylene glycol) and water as studied by differential scanning calorimetry. *J. Polym. Sci. Part B Polym. Phys.* **39**, 496-506 (2015).
7. Eagland, D., Crowther, N. J., & Butler, C. J. Interaction of poly(oxyethylene) with water as a function of molar mass. *Polymer* **34**, 2804-2808 (1993).
8. Tanford, C. Physical chemistry of macromolecules, New York: Wiley, 1961, p. 343-346.
9. Tam, C. M., & Tremblay, A. Y. Membrane pore characterization-comparison between single and multicomponent solute probe techniques. *J. Membrane Sci.* **57**, 271-287 (1991).
10. Zhi, C., Chen, F., Zeng, F., & Li, J. Preparation and characterization of the charged PDMS/Al_2O_3 composite nanofiltration membrane. *Desalination* **349**, 106-114 (2014).
11. Krasilnikov, O. V., & Bezrukov, S. M. Polymer partitioning from nonideal solutions into protein voids. *Macromolecules* **37**, 2650-2657 (2004).
12. Oukhaled, A. G., Biance, A. L., Pelta, J., Auvray, L., & Bacri, L. Transport of long neutral polymers in the semidilute regime through a protein nanopore. *Phys. Rev. Lett.* **108**, 088104 (2012).
13. Krasilnikov, O. V., Rodrigues, C. G., & Bezrukov, S. M. Single polymer molecules in a protein nanopore in the limit of a strong polymer-pore attraction. *Phys. Rev. Lett.* **97**, 018301 (2006).
14. Tsai, W. T., Yang, J. M., Lai, C. W., Cheng, Y. H., Lin, C. C., & Yeh, C. W. Characterization and adsorption properties of eggshells and eggshell membrane. *Bioresource Technol.* **97**, 488-493 (2006).
15. Tawarah, K. M., & Abu-Shamleh, H. M. A spectrophotometric determination of the formation

constants of the inclusion complexes of α- and β-cyclodextrins with the azonium and ammonium tautomers of methyl orange and methyl yellow. *J. Incl. Phenom. Mol. Recognit. Chem.* **11**, 29-40 (1991).

16. Kato, T., & Horikoshi, K. Colorimetric determination of γ-cyclodextrin. *Anal. Chem.* **56**, 1738-1740 (1984).
17. Cheng, X. Q., Liu, Y., Guo, Z., & Shao, L. Nanofiltration membrane achieving dual resistance to fouling and chlorine for "green" separation of antibiotics. *J. Membrane Sci.* **493**, 156-166 (2015).
